# Fine‐tuned photochromic sulfonylureas for optical control of beta cell Ca^2+^ fluxes

**DOI:** 10.1111/dme.15220

**Published:** 2023-09-21

**Authors:** Ann‐Kathrin Rückert, Julia Ast, Annie Hasib, Daniela Nasteska, Katrina Viloria, Johannes Broichhagen, David J. Hodson

**Affiliations:** ^1^ Leibniz‐Forschungsinstitut für Molekulare Pharmakologie Berlin Germany; ^2^ Institute of Metabolism and Systems Research (IMSR), and Centre of Membrane Proteins and Receptors (COMPARE) University of Birmingham Birmingham UK; ^3^ Oxford Centre for Diabetes, Endocrinology and Metabolism (OCDEM), NIHR Oxford Biomedical Research Centre, Churchill Hospital, Radcliffe Department of Medicine University of Oxford Oxford UK

**Keywords:** imaging, insulin, islet, photopharmacology, signal transduction

## Abstract

We previously developed, synthesized and tested light‐activated sulfonylureas for optical control of K_ATP_ channels and pancreatic beta cell activity in vitro and in vivo. Such technology relies on installation of azobenzene photoswitches onto the sulfonylurea backbone, affording light‐dependent isomerization, alteration in ligand affinity for SUR1 and hence K_ATP_ channel conductance. Inspired by molecular dynamics simulations and to further improve photoswitching characteristics, we set out to develop a novel push‐pull closed ring azobenzene unit, before installing this on the sulfonylurea glimepiride as a small molecule recipient. Three fine‐tuned, light‐activated sulfonylureas were synthesized, encompassing azetidine, pyrrolidine and piperidine closed rings. Azetidine‐, pyrrolidine‐ and piperidine‐based sulfonylureas all increased beta cell Ca^2+^‐spiking activity upon continuous blue light illumination, similarly to first generation JB253. Notably, the pyrrolidine‐based sulfonylurea showed superior switch OFF performance to JB253. As such, third generation sulfonylureas afford more precise optical control over primary pancreatic beta cells, and showcase the potential of pyrrolidine‐azobenzenes as chemical photoswitches across drug classes.

**Novelty Statement**

- Sulfonylureas containing a closed ring azobenzene allow optical control over beta cell activity.
- Sulfonylureas with small‐ and medium‐size closed rings display improved ON–OFF beta cell switching.
- Fine‐tuned photochromic sulfonylureas may be useful for the optical interrogation of K_ATP_ channel activity and beta cell function.
- More widely, the closed ring azobenzenes are applicable to other known small molecule photoswitches for receptors, ion channels and enzymes.

## 1 INTRODUCTION

Photopharmacology describes the use of light to target drug activity in space and time, allowing optical control over ion channels, G‐protein coupled receptors and enzyme activity (reviewed in^1^). In general, photopharmacology relies on modifying drugs with azobenzene photoresponsive units, which undergo isomerization following illumination,^2^ altering ligand‐target interactions.^3^ Previously, we and others have shown the broad utility of photopharmacology for the optical control of K_ATP_ channels,^4^, ^5^, ^6^ voltage‐dependent Ca^2+^ channels,^7^ protein kinase C,^8^ GPR40,^9^ guanylyl cyclase^10^ and glucagon‐like peptide‐1 receptors (GLP1R),^11^, ^12^ allowing interrogation of pancreatic beta cell signalling in vitro and in vivo (reviewed in^13^). Photopharmacology also allows optical control of endogenous cell machinery without the need for recombinant genetics, complex mouse models or cross‐talk from fluorophore reporters.

Despite this, there are a number of drawbacks with photopharmacology including: best performance in UV–visible wavelengths,^2^ which are non‐optimal for deep tissue manipulation; effects of illumination on chemical structure itself^12^; and lack of binary ON–OFF responses. Since sulfonylureas are relatively straightforward to synthesize, tolerate modification with azobenzene photoresponsive units, and are well validated over multiple studies,^4^, ^5^, ^6^, ^14^ they provide a good testbed to optimize photopharmacological approaches. For example, our original blue light‐activated sulfonylurea, **JB253**, could be modified with a heterocyclic aromatic unit to red‐shift responses from the 440 nm ➔ 520 nm range.^5^ Moreover, **JB253** was found to be stable even under intense UV illumination,^4^ unlike an allosteric GLP1R modulator that underwent rearrangement, presumably via an intramolecular Meisenheimer complex.^11^

In the present study, we reasoned that fine‐tuning the azobenzene electron‐donating push‐pull system might endow light‐activated sulfonylureas with better switching performance in the tissue‐setting. Based on recent molecular dynamics studies that modelled (active) *cis*‐**JB253** in the SUR1‐bound state,^3^ we noticed that the flexible *N*,*N*‐diethylamine occupies a gap that is relatively devoid of contact sites (Figure S1). We hypothesized that further reduction of ligand‐receptor contacts would allow higher probability of channel closure due to recruitment of the N‐terminal tail.^3^ Therefore, we decided to install different cyclic amines based on studies almost half a century ago showing their influence on 4‐aminoazobenzene electronic absorption spectra.^15^, ^16^, ^17^, ^18^

The consequent “closed‐ring” azobenzene‐sulfonylureas, spanning small‐large cyclic structures, were subject to detailed chemical characterization before in vitro testing in pancreatic islets. While all the novel light‐activated sulfonylureas showed photoswitching of beta cell Ca^2+^ fluxes, those with a medium‐sized pyrrolidine ring evoked the best ON–OFF responses. Thus, fine‐tuned light‐activated sulfonylureas demonstrate the utility of pyrrolidine rings for optical interrogation of beta cell function, with broad applicability to other drug classes.

## 2 RESULTS

### 2.1 Synthesis of fine‐tuned light‐activated sulfonylureas

**FIGURE 1:**
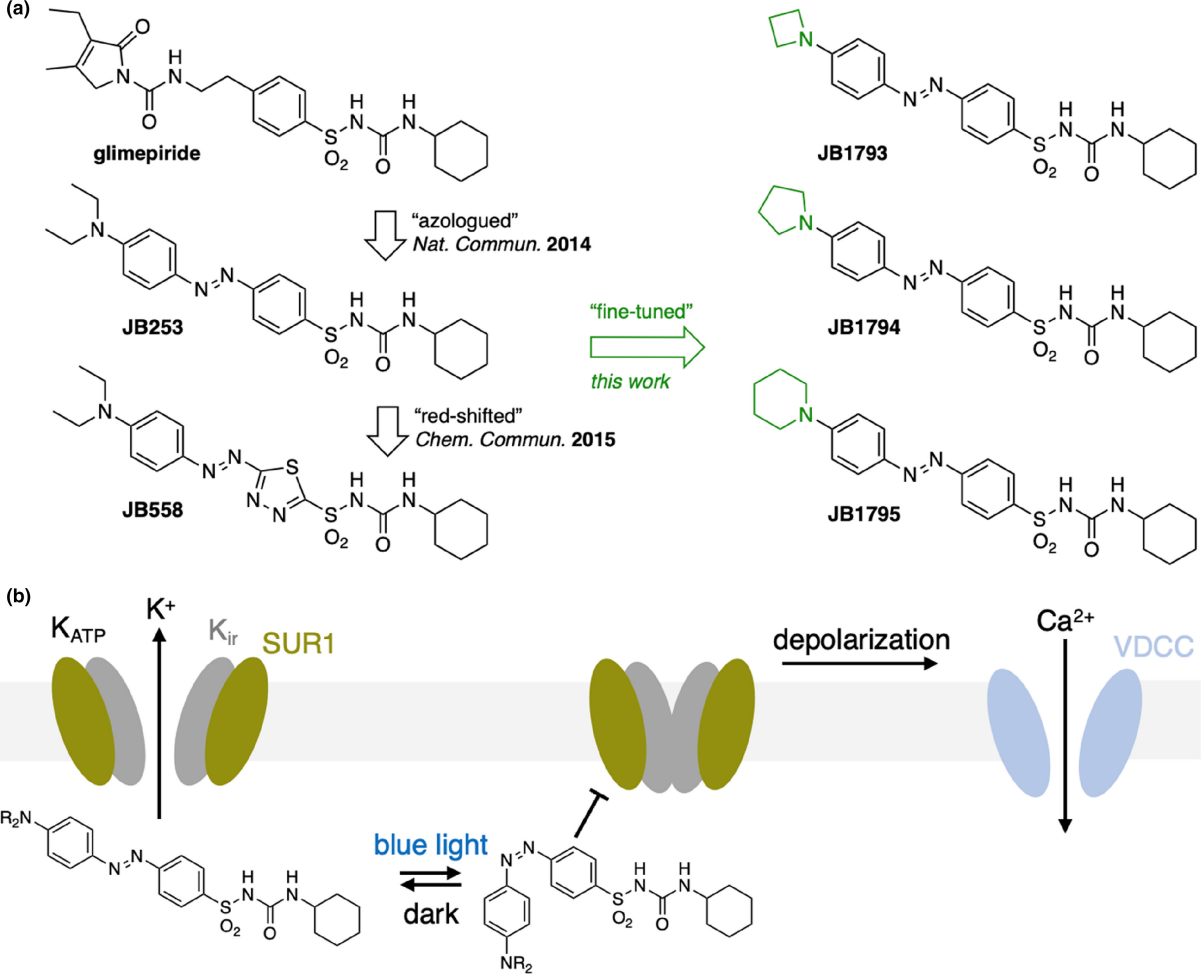
Design and logic of controlling K_ATP_ channels with light. (a) The K_ATP_ channel blocker glimepiride, a sulfonylurea, is endowed with a blue light sensitive azobenzene photoswitch to obtain **JB253**, which was further optimized to respond to yellow‐green light as its congener **JB558**. Herein, we describe fine‐tuning of **JB253** by replacing the *N*,*N*‐diethyl groups with ring structures of various sizes, that is, four‐membered azetidine (**JB1793**), five‐membered pyrrolidine (**JB1794**) and six‐membered piperidine (**JB1795**). (b) K_ATP_ channels comprise four K_ir_ and four SUR1 subunits and are constitutively open, allowing K^+^ efflux. Azobenzene sulfonylureas are unable to block current flow in one state (*trans*, left), but when switched to *cis* (right) with blue light, the channel is blocked. This leads to membrane depolarization and activation of voltage dependent Ca^2+^ channels, leading to Ca^2+^influx and insulin secretion.

We previously reported **JB253** and **JB558**, sulfonylureas activated by blue and green‐yellow light.^4^, ^5^ In this study, three novel sulfonylurea‐containing azobenzene photoresponsive units were designed (Figure 1a) by further fine‐tuning the **JB253** scaffold. As such, the *N*,*N*‐diethyl amino group (**JB253**) was formally closed to a pyrrolidine (**JB1794**) and the ring size was both reduced and enlarged to an azetidine (**JB1793**) and a piperidine (**JB1795**), respectively. We anticipated that this small change would not have an effect on switching kinetics and wavelength sensitivity, but might change the interaction with its target, the K_ATP_ channel. When toggled to its *cis*‐isomer using blue light, **JB253** is able to block K^+^ efflux from K_ATP_ channels in pancreatic beta cells (Figure 1b).

**FIGURE 2:**
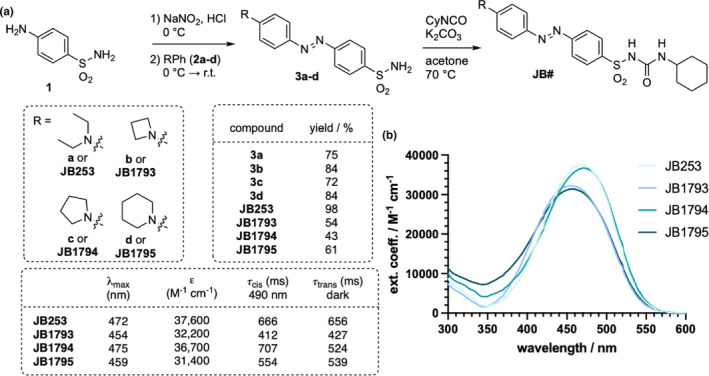
Synthesis and characterization of fine‐tuned azobenzene sulfonylureas. (a) Commencing with sulfanilamide (1), azobenzene sulfonylureas are obtained in a two‐step synthetic sequence, by first diazotization and trapping the resulting diazonium salt with an alkylated aniline. Installation of the sulfonylurea is achieved using cyclohexyl isocyanate, yielding **JB#** photoswitches. Maximal absorbance wavelengths and switching kinetics in response to 490 nm light and dark‐relaxation denoted in the bottom table. (b) UV/Vis spectra of **JB253**, **JB1793**, **JB1794** and **JB1795** in DMSO show maximal wavelength of absorption shift and differences in extinction coefficient.

Synthetically, the azobenzene unit was installed using sulfanilamide, with sodium nitrite in hydrochloric acid as the nitrosylating agent, and the prepared diazonium salt was quenched in situ to yield the azobenzene (Figure 2a, and see Data S1). For this, several anilines were used as nucleophiles to yield azobenzene sulfonamides **3a‐d**.^13^ The sulfonylurea unit was installed by an addition reaction between cyclohexyl isocyanate and the respective sulfonamide **3** to give the sulfonylurea‐containing azobenzenes **JB1793‐5**. All compounds were HPLC purified and homogeneity was assessed by ^1^H NMR (See Data S1).

### 2.2 Absorption spectra and switching kinetics of fine‐tuned light‐activated sulfonylureas

The absorption maxima λ_max_ of **JB253** and **JB1793‐5** were determined by UV/Vis (Figure 2b) in DMSO. λ_max_ of the *N,N*‐diethylamine‐containing azobenzene **JB253** (*λ*_max_ = 472 nm) was similar to the pyrrolidine‐containing azobenzene **JB1794 (** *λ*_max_ = 475 nm), which possesses a formally closed ring. With decreasing (**JB1793**) or increasing (**JB1795**) ring size, the absorption maxima are blue‐shifted to *λ*_max_ = 454 nm and *λ*_max_ = 459 nm, respectively.

We also determined the extinction coefficient by first measuring ^1^H qNMR with 1,3,5‐trimethoxybenzene serving as an internal standard in DMSO‐d_6_ (see Data S1). Aliquots were then taken and subjected to UV/Vis spectroscopy to obtain extinction values (ε) according to Lambert–Beer's Law (Figure 2b). **JB253** shows an *ε* = 37,600 M^−1^ cm^−1^, which is close to **JB1794** with *ε* = 36,700 M^−1^ cm^−1^. In contrast, **JB1793** and **JB1795** showed smaller extinction coefficients, being *ε* = 32,200 M^−1^ cm^−1^ and *ε* = 31,400 M^−1^ cm^−1^, respectively.

Red‐shifted azobenzene photoresponsive units like **JB253** are solely present in the *trans*‐state in the absence of light, with the *cis*‐state obtained by irradiation with visible light. Photoswitching was therefore assessed using a monochromator to deliver 490 nm light, with peak responses observed at *λ* = 474 nm, *λ* = 474 nm, *λ* = 453 nm and *λ* = 458 nm for **JB253**, **JB1793**, **JB1794** and **JB1795**, respectively. Measuring multiple cycles between irradiation ON and OFF, we exponentially fitted the corresponding curves to obtain *τ*_cis_ and *τ*_trans_, in increasing order for *τ*_cis_: **JB1793**: *τ*_cis_ = 412 ms; *τ*_trans_ = 427 ms; **JB1795**: *τ*_cis_ = 554 ms; *τ*_trans_ = 539 ms; **JB253**: *τ*_cis_ = 666 ms; *τ*_trans_ = 656 ms; **JB1794**: *τ*_cis_ = 707 ms; *τ*_trans_ = 524 ms (Figure 2a,b, see Data S1).

### 2.3 Activity of fine‐tuned light‐activated sulfonylureas

Pancreatic islets were used as a relevant testbed to assess the photoswitching profile of **JB253**, **JB1793**, **JB1794** and **JB1795**. Insulin‐secreting beta cells respond to high blood glucose levels by increasing the ATP/ADP ratio, which leads to closure of K_ATP_ channels, membrane depolarization, opening of voltage‐dependent Ca^2+^ channels and Ca^2+^‐dependent insulin secretion,^19^, ^20^ alongside contributions from the PEP cycle.^21^ Thus, Ca^2+^ imaging provides a convenient proxy to longitudinally and dynamically screen K_ATP_ channel activity using trappable dyes.

**FIGURE 3:**
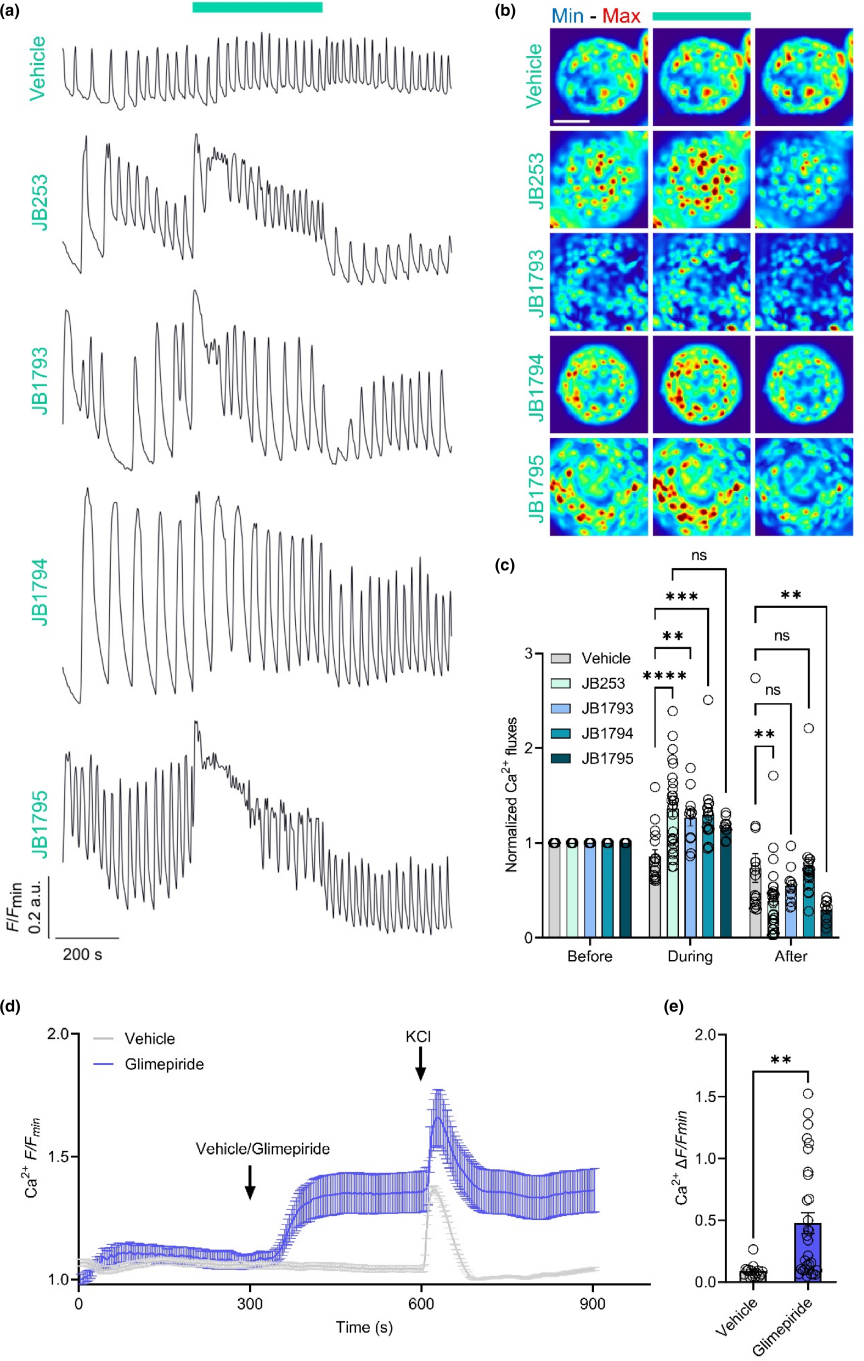
Fine‐tuning photochromic sulfonylureas improves optical control over beta cell Ca^2+^ fluxes. (a) Representative Ca^2+^ traces from vehicle‐, **JB253**, **JB1793**, **JB1794** and **JB1795** ‐treated islets (8 mM glucose), showing Ca^2+^‐spiking activity before, during and after continuous blue light illumination at 470 nm. Note the absence of effect in vehicle‐treated islets, confirming that blue light per se does not trigger Ca^2+^ spikes (vehicle, *n* = 16 islets, 5 animals; **JB253**, *n* = 27 islets, 8 animals; **JB1793**, *n* = 11 islets, 5 animals; **JB1794**, *n* = 18 islets, 8 animals; **JB1795**, *n* = 9 islets, 5 animals). (b) As for (a), but representative images showing changes in Ca^2+^ fluxes (scale bar = 50 μm). (c) Summary bar graph showing stimulation of Ca^2+^ fluxes by **JB253**, **JB1793**, **JB1794** and **JB1795** in response to blue light illumination (two‐way ANOVA with Tukey post‐hoc test). After illumination, only **JB1793** and **JB1794** show similar Ca^2+^ levels to vehicle controls. (d, e) Glimepiride, but not vehicle control, leads to a large increase in intracellular Ca^2+^ levels at 8 mM glucose, shown by mean traces (D), as well as Δ*F/F*_*min*_ (E) (unpaired *t*‐test) (*n* = 16–30 islets, 5 animals). **JB253**, **JB1793**, **JB1794**, **JB1795** and glimepiride were applied at 50 μM, while vehicle contained DMSO 0.1%. Bar graphs show individual data points and mean ± SEM.

Islets isolated from C57BL/6J mice were loaded with the Ca^2+^ dye, Fluo8, before application of either vehicle (0.1% DMSO) or 50 μM **JB253**, **JB1793**, **JB1794** or **JB1795**, and timelapse spinning disk confocal microscopy. Islets were maintained at 8 mM glucose, which has previously been shown to be optimal for sulfonylurea activity in beta cells.^22^ To assess **JB253**, **JB1793**, **JB1794** and **JB1795** activity, blue light illumination (470 nm) was delivered as 150 ms pulses at 0.5 Hz, which allows Fluo8 excitation, before continuous illumination to trigger compound photoactivation. As expected, under this protocol, no effects of vehicle or continuous illumination could be detected on Ca^2+^ spiking activity (Figure 3a,b).

Confirming previous results, photoactivation of **JB253** evoked a significant increase in Ca^2+^ fluxes, determined over the duration of illumination using area‐under‐the‐curve (AUC) normalized to vehicle (Figure 3a–c). Following cessation of continuous blue light, Ca^2+^−spiking amplitude rapidly decreased, rebounding below initial levels before slowly recovering to pre‐stimulation levels, probably reflecting voltage‐inactivation of Ca^2+^ channels (Figure 3a–c). **JB1793** and **JB1794** showed similar photocontrol of Ca^2+^ amplitude, but in contrast to **JB253**, a better ON–OFF response was observed, without the large negative rebound in Ca^2+^ amplitude (Figure 3a–c). Of note, **JB1795** displayed similar photoswitching responses to **JB253** (Figure 3a–c).

Glimepiride‐alone (50 μM) led to a large increase in intracellular Ca^2+^ concentration, rather than changes in Ca^2+^ oscillation frequency (Figure 3d,e). This effect was expected, since glimepiride has an IC_50_ for Kir6.2/SUR1 inhibition of ~3.0–5.0 nM, whereas **JB253** was shown to have 1000‐fold lower affinity for SUR1, lending itself to superior K_ATP_ channel switching in the trans‐ and cis‐ states.^4^ Together these results show that closing the *N*,*N*‐diethyl amino group to form pyrrolidine ring‐containing **JB1794** imparts better photoswitching on light‐activated sulfonylureas, with small azetidine **JB1793**, but not large piperidine **JB1795** ring sizes, favouring more binary optical control of cell activity.

## 3 DISCUSSION

In the present study, we set out to generate fine‐tuned light‐activated sulfonylureas with superior photoswitching performance for the spatiotemporal control of pancreatic beta cell Ca^2+^ fluxes. To do this, three novel sulfonylureas (**JB1793**, **JB1794** and **JB1795**) were produced with azetidine, pyrrolidine and piperidine closed rings replacing the *N*,*N*‐diethyl amino group to form a push‐pull azobenzene. Sulfonylureas **JB1793**, **JB1794** and **JB1795** were tested head–head against the well characterized first generation light‐activated sulfonylurea **JB253**, and found to increase intracellular Ca^2+^ fluxes to a similar extent in response to blue light illumination. However, sulfonylureas **JB1793** and **JB1794**—spanning small‐ to moderate‐sized rings—showed superior photoswitching performance, without the characteristic negative rebound in Ca^2+^ fluxes observed with **JB253** following cessation of blue light illumination. Demonstrating an important role for ring size in azobenzene back relaxation, the piperidine‐containing **JB1795** showed the largest negative rebound.

Interactions between sulfonylureas and SUR have been extensively studied by means of pharmacology, mutational scans and structural work.^23^, ^24^, ^25^, ^26^, ^27^, ^28^ In silico‐predicted binding modes of **JB253** to SUR1 have been reported,^3^ which suggest that optimizing the **JB253** scaffold might be beneficial at the *N,N*‐diethylamine group. We decided to lock the flexible ethyl groups to a ring structure, and to gain more insight into our approach, we synthesized three different ring sizes (4, 5 and 6‐membered). Using this approach, we found that interactions between SUR1:**JB1793** and SUR1:**JB1794**, that is, small‐medium ring sizes, were preferable for optical control of Ca^2+^ fluxes when switching between *cis‐* and *trans‐* using 470 nm and dark conditions, respectively. This reflects the optimal situation of the nitrogen‐containing ring engaging with SUR1 for channel closure.

There are a number of limitations that need to be considered in the present study. Firstly, we were unable to provide photostationary states measures of **JB253**, **JB1793**, **JB1794** and **JB1795**, which has proved difficult for red‐shifted, dark relaxing azobenzenes, presumably since illumination powers required *ex cellulo* could not be easily introduced into NMR instrument(s) used. Secondly, we focused our screening efforts on beta cell Ca^2+^ fluxes, and did not measure insulin secretion, which is less amenable (and robust) to head–head comparison of photoswitching responses across multiple ligands. While K_ATP_ channel‐driven Ca^2+^ fluxes are expected to translate into insulin secretion, this should be confirmed in future experiments, for instance by: (1) batch incubation/perifusion‐based insulin assays in the presence of JB1793‐1795 ± light^4^, ^5^; or (2) simultaneous measurement of Ca^2+^ fluxes and insulin secretion using, for example, fluorescent Zn^2+^ probes to measure Zn^2+^ co‐released with insulin.^14^, ^29^, ^30^ Lastly, SUR1‐binding affinity of **JB1793**, **JB1794** and **JB1795** were not examined using competition assays with [^3^H]glibenclamide because fastback‐relaxation might cause diffusion.^4^ This may be examined in the future by molecular dynamics simulations, although the changes in Ca^2+^ fluxes shown here provide a reasonably accurate downstream indicator of K_ATP_ channel activity.

In summary, we show that “closing the ring” endows light‐activated sulfonylureas with superior ON–OFF photoswitching performance in pancreatic beta cells. Such tools allow reliable and robust optical control of endogenous K_ATP_ channel activity and Ca^2+^ fluxes without the need for genetic recombination. We expect that the design template here will be applicable to a broad range of other small molecule ligands that rely on azobenzene photoresponsive elements to optically control ion channels, GPCRs and enzymes.

## 4 METHODS

### 4.1 Chemical synthesis

All synthetic protocols and characterization can be found in the Data S1.

### 4.2 Ethics

Animal studies were regulated by the Animals (Scientific Procedures) Act 1986 of the UK (Personal Project Licences P2ABC3A83 and PP1778740). Approval was granted by the University of Birmingham's and University of Oxford's Animal Welfare and Ethical Review Bodies (AWERB). All ethical guidelines were adhered to while carrying out this study.

### 4.3 Mice

Male and female C57BL/6J mice, 7–10 weeks old, were used as wild‐type tissue donors. Mice were socially housed in specific‐pathogen free conditions at Biomedical Services Unit, University of Birmingham, under a 12 h light–dark cycle with ad libitum access to food and water. Relative humidity was 55 ± 10% and temperature 21 ± 2°C.

### 4.4 Islet isolation

Mice were humanely culled using a schedule‐1 method before confirmation of death. Collagenase NB 8 (Serva) was diluted in RPMI 1640 (Gibco) at 1 mg/mL and injected into the bile duct before dissection of the pancreas and storage on wet ice pending digestion. Pancreases were digested in a water bath at 37°C for 12 min. Following washing, islets were gradient‐separated using Histopaque‐1119 and 1083 (Sigma‐Aldrich), before hand‐picking and culture. Islets were cultured in RPMI 1640 supplemented with 10% fetal bovine serum (FBS, Gibco), 100 units/mL penicillin and 100 μg/mL streptomycin (Sigma‐Aldrich), at 37°C and 5% CO_2_.

### 4.5 Multicellular Ca^2+^ imaging

Islets were loaded with Fluo 8 (AAT Bioquest, cat. no. 20494) in HEPES‐bicarbonate buffer containing (in mmol/L) 120 NaCl, 4.8 KCl, 24 NaHCO_3_, 0.5 Na_2_HPO_4_, 5 HEPES, 2.5 CaCl_2_, 1.2 MgCl_2_ and supplemented with 8 mM *D*‐glucose. Ca^2+^ fluxes were measured using a spinning disk microscope comprised of a Nikon Ti‐E frame, 10×/0.3 NA air objective, North 89 LDI laser bank and CrestOptics V2 X‐light spinning disk unit. Excitation was delivered at *λ* = 470 nm, with emission collected at *λ* = 500–550 nm. Intracellular Ca^2+^ traces were normalized as F/F_min_, where F is fluorescence at any given time point, and F_min_ is mean minimum fluorescence. To calculate photoswitching efficiency for each compound, AUC was calculated at each timepoint and then normalized to values before illumination. **JB253**, **JB1793**, **JB1794, JB1795** and glimepiride were applied to islets at 50 μM.

### 4.6 Statistics and reproducibility

GraphPad Prism 9 (version 9.2.0) was used for statistical analysis. Multiple interactions were determined using two‐way ANOVA with Tukey post‐hoc test. Error bars represent mean ± S.E.M. and a *p*‐value less than 0.05 was considered significant: *p < 0.05; **p < 0.01; ***p < 0.001, ****p < 0.0001.

## CONFLICT OF INTEREST STATEMENT

J.B. and D.J.H. receive licensing revenue from Celtarys Research for provision of chemical probes. J.B. and D.J.H. hold a patent concerning photoswitchable sulfonylureas (WO2016059093A1). J.A. is currently an employee of Novo Nordisk. The remaining authors declare no competing interests.

## Supporting information


Data S1.

## Data Availability

The data that support the findings of this study are available from the corresponding author upon reasonable request.
