# Supplementary material for: Fine‐tuned photochromic sulfonylureas for optical control of beta cell Ca2+ fluxes
Source: Diabet Med. 2023 Sep 21;40(12):e15220. doi: 10.1111/dme.15220 (PMC10947021; doi:10.1111/dme.15220)
Supplement: Supplementary file 1 — Data S1. [file DME-40-0-s001.docx]

Supplemental Information for:

**Fine-tuned photochromic sulfonylureas for optical control of beta cell Ca^2+^ fluxes**

Ann-Kathrin Rückert^1,^^, Julia Ast^2,^^, Annie Hasib^2^, Daniela Nasteska^3^, Katrina Viloria^2,3^, Johannes Broichhagen^1^* and David J. Hodson^2,3^*

^1^ Leibniz-Forschungsinstitut für Molekulare Pharmakologie, Berlin, Germany.

^2^ Institute of Metabolism and Systems Research (IMSR), and Centre of Membrane Proteins and Receptors (COMPARE), University of Birmingham, Birmingham, UK.

^3^ Oxford Centre for Diabetes, Endocrinology and Metabolism (OCDEM), NIHR Oxford Biomedical Research Centre, Churchill Hospital, Radcliffe Department of Medicine, University of Oxford, Oxford, UK.

^^^joint first authors

*Correspondence should be addressed to:

[david.hodson@ocdem.ox.ac.uk](mailto:david.hodson@ocdem.ox.ac.uk), [broichhagen@fmp-berlin.de](mailto:broichhagen@fmp-berlin.de)

**Table of Contents**

[1. General 3](#_Toc139620027)

[2. Synthesis 4](#_Toc139620028)

[2.1. 1-Phenylazetidine 4](#_Toc139620029)

[2.2. General procedure A for azobenzene synthesis via diazotation 4](#_Toc139620030)

[2.3. (E)-4-((4-(Diethylamino)phenyl)diazenyl)benzenesulfonamide (3a) 4](#_Toc139620031)

[2.4. (E)-4-((4-(Azetidin-1-yl)phenyl)diazenyl)benzenesulfonamide (3b) 5](#_Toc139620032)

[2.5. (E)-4-((4-(Pyrrolidin-1-yl)phenyl)diazenyl)benzenesulfonamide (3c) 5](#_Toc139620033)

[2.6. (E)-4-((4-(Piperidin-1-yl)phenyl)diazenyl)benzenesulfonamide (3d) 5](#_Toc139620034)

[2.7. General procedure B for sulfonylurea installation 6](#_Toc139620035)

[2.8. (E)-N-(Cyclohexylcarbamoyl)-4-((4-(diethylamino)phenyl)diazenyl)- benzenesulfonamide (4a or JB253) 6](#_Toc139620036)

[2.9. (E)-N-(Cyclohexylcarbamoyl)-4-((4-(azetidin-1-yl)phenyl)diazenyl)- benzenesulfonamide (4b or JB1793) 6](#_Toc139620037)

[2.10. €-N-(Cyclohexylcarbamoyl)-4-((iazinylnedin-1-yl)pheniazinylnyl)- benzenesulfonamide (4c) 7](#_Toc139620038)

[2.11. (E)-N-(Cyclohexylcarbamoyl)-4-((4-(piperidin-1-yl)phenyl)diazenyl)- benzenesulfonamide (4d or JB1794) 7](#_Toc139620039)

[3. NMR spectra 8](#_Toc139620040)

[3.1. 1-Phenylazetidine 8](#_Toc139620041)

[3.2. (E)-4-((4-(Diethylamino)phenyl)diazenyl)benzenesulfonamide (3a) 9](#_Toc139620042)

[3.3. (E)-4-((4-(Azetidin-1-yl)phenyl)diazenyl)benzenesulfonamide (3b) 10](#_Toc139620043)

[3.4. (E)-4-((4-(Pyrrolidin-1-yl)phenyl)diazenyl)benzenesulfonamide (3c) 11](#_Toc139620044)

[3.5. (E)-4-((4-(Piperidin-1-yl)phenyl)diazenyl)benzenesulfonamide (3d) 12](#_Toc139620045)

[3.6. (E)-N-(Cyclohexylcarbamoyl)-4-((4-(diethylamino)phenyl)diazenyl)- benzenesulfonamide (4a or JB253) 13](#_Toc139620046)

[3.7. (E)-N-(Cyclohexylcarbamoyl)-4-((4-(azetidin-1-yl)phenyl)diazenyl)- benzenesulfonamide (4b or JB1793) 14](#_Toc139620047)

[3.8. (E)-N-(Cyclohexylcarbamoyl)-4-((4-(pyrrolidin-1-yl)phenyl)diazenyl)- benzenesulfonamide (4c or JB1794) 15](#_Toc139620048)

[3.9. (E)-N-(Cyclohexylcarbamoyl)-4-((4-(piperidin-1-yl)phenyl)diazenyl)- benzenesulfonamide (4d or JB1795) 17](#_Toc139620049)

[4.1. (E)-N-(Cyclohexylcarbamoyl)-4-((4-(diethylamino)phenyl)diazenyl)- benzenesulfonamide (4a or JB253) 19](#_Toc139620050)

[4.2. (E)-N-(Cyclohexylcarbamoyl)-4-((4-(azetidin-1-yl)phenyl)diazenyl)- benzenesulfonamide (4b or JB1793) 19](#_Toc139620051)

[4.3. (E)-N-(Cyclohexylcarbamoyl)-4-((4-(pyrrolidin-1-yl)phenyl)diazenyl)- benzenesulfonamide (4c or JB1794) 20](#_Toc139620052)

[4.4. (E)-N-(Cyclohexylcarbamoyl)-4-((4-(piperidin-1-yl)phenyl)diazenyl)- benzenesulfonamide (4d or JB1795) 20](#_Toc139620053)

[5.1. (E)-N-(Cyclohexylcarbamoyl)-4-((4-(diethylamino)phenyl)diazenyl)- benzenesulfonamide (4a or JB253) 21](#_Toc139620054)

[5.2. (E)-N-(Cyclohexylcarbamoyl)-4-((4-(azetidin-1-yl)phenyl)diazenyl)- benzenesulfonamide (4b or JB1793) 21](#_Toc139620055)

[5.3. (E)-N-(Cyclohexylcarbamoyl)-4-((4-(pyrrolidin-1-yl)phenyl)diazenyl)- benzenesulfonamide (4c or JB1794) 22](#_Toc139620056)

[5.4. (E)-N-(Cyclohexylcarbamoyl)-4-((4-(piperidin-1-yl)phenyl)diazenyl)- benzenesulfonamide (4d or JB1795) 22](#_Toc139620057)

[6. References 22](#_Toc139620058)

# General

All chemical reagents and anhydrous solvents for synthesis were purchased from commercial suppliers (Sigma-Aldrich, VWR, Carl Roth, Fluorochem) and were used without further purification if not stated otherwise.

NMR spectra were recorded at ambient temperature in deuterated solvents on a Bruker AV III 600 MHz spectrometer. ^1^H and ^13^C chemical shifts were referenced to residual solvent peaks. ^13^C NMR spectra were hydrogen decoupled. Multiplicities are abbreviated as follows: s = singlet, d = doublet, t = triplet, q = quartet, br = broad, m = multiplet. Chemical shifts are reported in ppm and coupling constants are reported in Hz. Spectra are reported based on appearance, not on theoretical multiplicities derived from structural information.

Preparative and semi-preparative HPLC was performed on an Agilent 1260 Infinity II LC System equipped with columns as followed: preparative column: Reprospher 100 C18 columns (10 µm: 50 x 30 mm at 20 mL/min flow rate; semi-preparative column: 5 µm: 250 x 10 mm at 4 mL/min flow rate. Eluents A (0.1% TFA in H_2_O) and B (0.1% TFA in MeCN) were applied as a linear gradient. Peak detection was performed at maximal absorbance wavelength.

LC-MS was performed on an Agilent 1260 Infinity HPLC System, MS-Agilent 1100 Series, Type: 1946D, Model: SL, equipped with a Agilent Zorbax Eclipse Plus C18 (100 x 4.6 mm, particle size 3.5 micron) RP column. The typical gradient was from 10% B for 0.5 min 🡪 gradient to 95% B over 5 min 🡪 95% B for 0.5 min 🡪 gradient to 99% B over 1 min with 0.8 mL/min flow.

High resolution mass spectrometry analyses were carried out using the spectrometer Agilent Technologies 6530 Accurate Mass Q-ToF LC/MS linked to Agilent Technologies HPLC 1260 Infinity II equipped with column: Thermo Accuore RP-MS; Particle Size: 2.6 µm; Dimension: 30 x 2.1 mm and using following Gradient: Eluent A: Water with 0.1% FA; Eluent B: Acetonitrile with 0.1% FA. 0.00 min 95% A, 0.1 min 95% A, 1.0 min 1% A, 3.5 min Stoptime; 1.3 min Posttime; Flow rate: 0.8 ml/min^−1^.

Ultraviolet/Vis spectra were recorded on a Jasco V-550 UV/Vis Spectrophotometer using Helma Suprasil precision cuvettes (10 mm and 3 mm light path).

Irradiation of the samples for isomerization kinetics was performed using the CoolLED pE-4000 Universal Light Source.

# Synthesis

## 1-Phenylazetidine


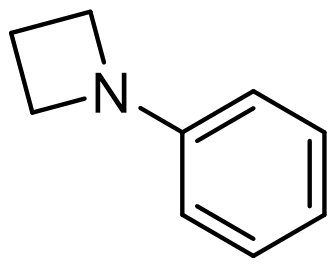


A flame dried schlenk flask was filled with iodobenzene (510 mg, 274 *µ*L, 2.5 mmol, 1.0 equiv.), Cs_2_CO_3_ (1.9 g, 5.9 mmol, 2.4 equiv.) XPhos Pd G2 (315 mg, 0.4 mmol, 0.15 equiv.), azetidine (166 mg, 200 *µ*L, 2.9 mmol, 1.2 equiv.) and dry 1,4-dioxane (12 mL). The solution was stirred for 4 h at 80 °C. The mixture was cooled to 60 °C to add another portion of azetidine (166 mg, 200 *µ*L, 2.9 mmol, 1.2 equiv.). After stirring the mixture for 17 h at 70 °C and 17 h at r.t., 1-phenylazetidine was extracted with DCM (120 mL) against water (120 mL). The aqueous layer was washed with DCM and the combined organic layer was washed with water and brine and dried over MgSO_4_. After filtering, all volatiles were removed *in vacuo*. Column chromatography yieled 82% () of the desired product as a brown oil.

**^1^H NMR** (600 MHz, DMSO-d_6_): *δ* [ppm] = 7.24 (q, *J* = 5.3 Hz, 2H), 6.76 (t, *J* = 7.3 Hz, 1H), 6.49 (dd, *J* = 1.0 Hz, 2H), 3.90 (t, *J* = 7.2 Hz, 4H), 2.39 (m, 2H).

**^13^C NMR** (150 MHz, DMSO-d_6_): *δ* [ppm] = 152.4, 129.0, 117.5, 111.5, 52.6,17.1.

**HR-MS** (ESI): calc. for C_9_H_12_N^+^ [M+H]^+^: 134.0964, found:134.0963.

**R_t_** (LC-MS); MeCN/H_2_O/formic acid = 1/99/0.1 to 95/5/0.1 over 7 min) = 2.496 min

## General procedure A for azobenzene synthesis *via* diazotation

The azobenzenes (**3a-d**) were synthesized adopted form a procedure described in the literature.^1^ Sulfanilamide (**1**) (117 mg, 0.68 mmol, 1.0 equiv.) was dissolved in 2.4 M HCl and cooled to 0 °C using an ice bath. A 2.3 M solution of sodium nitrite (0.6 mL, 0.82 mmol, 1.2 equiv.) was added dropwise under vigorous stirring while the mixture turned pale yellow. After stirring the solution for another 10 minutes, a mixture of the aniline (**2a-d**) (0.68 mmol, 1.0 equiv.) dissolved in MeOH:1 M NaOAc (1:20) and were added. The resulting dark red suspension was stirred for an additional hour allowing to warm to r.t.. The crude product was extracted with EtOAc (3 x 20 mL) and the organic layers were combined, washed with water and brine and dried over MgSO_4_. All volatiles were removed *in vacuo*.

## (*E*)-4-((4-(Diethylamino)phenyl)diazenyl)benzenesulfonamide (3a)


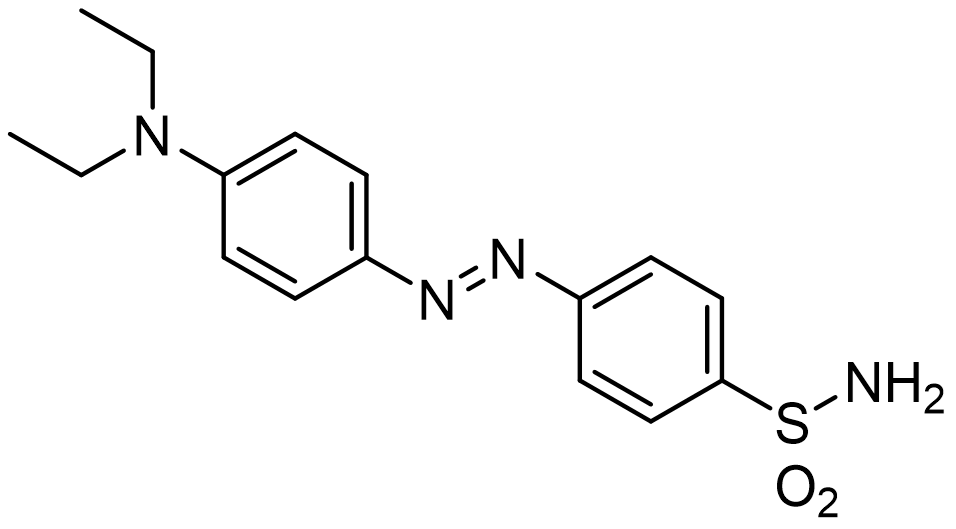


Column chromatography (1:1 EtOAc:cyclohexane) yielded 75% (170 mg) of the desired product as a red powder. For characterization, 20 mg was subjected to HPLC for purification.

**^1^H NMR** (600 MHz, DMSO-d_6_): *δ* (ppm) = 7.95 (d, *J* = 8.6 Hz, 2H),7.88 (d, *J* = 8.6 Hz, 2H), 7.81 (d, *J* = 9.2 Hz, 2H), 7.43 (s, 2H), 6.83 (d, *J* = 9.2 Hz, 2H), 3.48 (q, *J* = 7.1 Hz, 4H), 1.16 (t, *J* = 7.1 Hz, 6H).

**^13^C NMR** (150 MHz, DMSO-d_6_): *δ* (ppm) = 154.2, 150.8, 143.8, 142.1, 126.9, 125.7, 121.9, 111.1, 44.1, 12.5.

**HR-MS** (ESI): calc. for C_16_H_21_N_4_O_2_S^+^ [M+H]^+^: 333.1380, found: 333.1427.

**R_t_** (LC-MS): MeCN/H_2_O/formic acid = 10/90/0.1 to 95/5/0.1 over 7 min) = 4.730 min.

**λ_max_** (LC-MS) = 466 nm

## (*E*)-4-((4-(Azetidin-1-yl)phenyl)diazenyl)benzenesulfonamide (3b)


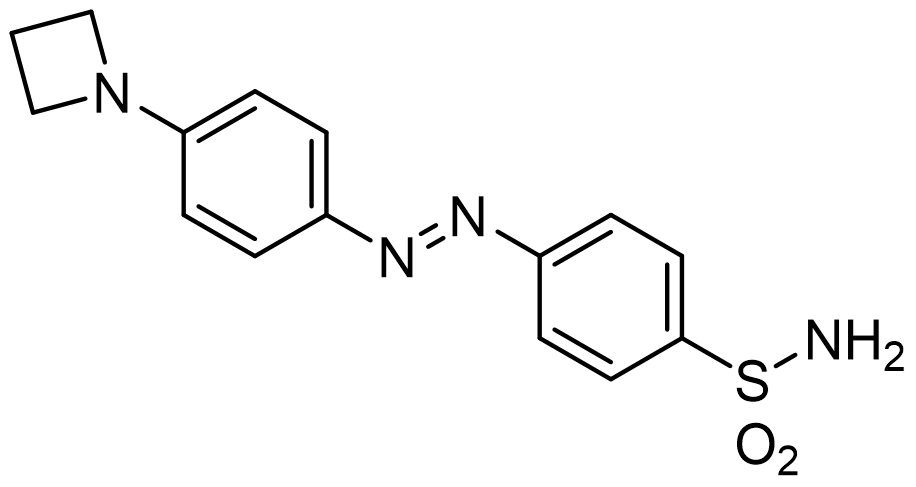


The crude product was purified via HPLC yielding 84% (181 mg) of the desired product as a red powder.

**^1^H NMR** (600 MHz, DMSO-d_6_): *δ* (ppm) = 7.95 (d, *J*= 8.5 Hz, 2H), 7.89 (d, *J*= 8.5 Hz, 2H), 7.82 (d, *J*= 8.8 Hz, 2H), 7.44 (s, 2H), 4.02 (t, *J*= 7.4 Hz, 4H), 2.39 (m, 2H).

**^13^C NMR** (150 MHz, DMSO-d_6_): *δ* (ppm) = 154.1, 153.8, 144.1, 143.1, 126.9, 125.3, 122.0, 110.3, 51.4, 15.9.

**HR-MS** (ESI): calc. for C_15_H_17_N_4_O_2_S^+^ [M+H]^+^: 317.1067, found: 317.1072.

**R_t_** (LC-MS); MeCN/H_2_O/formic acid=10/90/0.1 to 95/5/0.1 over 7 min) = 4.298 min.

## (*E*)-4-((4-(Pyrrolidin-1-yl)phenyl)diazenyl)benzenesulfonamide (3c)


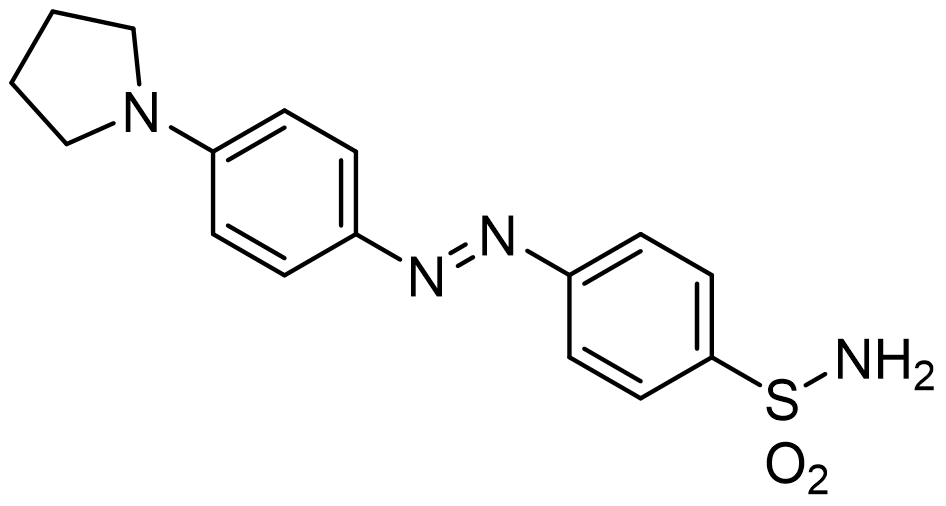


Column chromatography (1:1 EtOAc:cyclohexane to 2:1 EtOAc:cyclohexane) yielded 72% (162 mg) of the desired product as a red powder. For characterization, 20 mg was subjected to HPLC for purification.

**^1^H NMR** (600 MHz, DMSO-d_6_): *δ* (ppm) = 7.95 (d, *J*= 8.7 Hz, 2H), 7.89 (d, *J*= 8,8 Hz, 7.83 (d, *J*= 9,1 Hz, 2H), 7.43 (s, 2H), 6.70 (d, *J*= 9.2 Hz, 2H), 3.39 (m, 4H), 2.00 (m, 4H).

**^13^C NMR** (150 MHz, DMSO-d_6_): *δ* (ppm) = 154.7, 151.0, 144.3, 142.9, 127.4, 126.1, 122.4, 112.2, 48.0, 25.4.

**HR-MS** (ESI): calc. for C_16_H_19_N_4_O_2_S^+^ [M+H]^+^: 331.1223, found: 331.1232.

**R_t_** (LC-MS); MeCN/H_2_O/formic acid=10/90/0.1 to 95/5/0.1 over 7 min) = 4.585 min.

**λ_max_** (LC-MS) = 474 nm

**λ_max_** (LC-MS) = 466 nm

## (*E*)-4-((4-(Piperidin-1-yl)phenyl)diazenyl)benzenesulfonamide (3d)


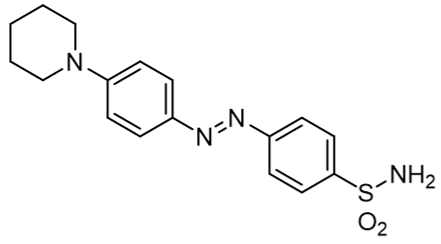


Column chromatography (1:1 EtOAc:cyclohexane) yielded 84% (199 mg) of the desired product as a red powder. For characterization, 20 mg was subjected to HPLC for purification.

**^1^H NMR** (600 MHz, DMSO-d_6_): *δ* (ppm) = 7.96 (d, *J*= 8.6 Hz, 2H), 7.91 (d, *J*= 8.6 Hz, 2H), 7.82 (d, *J*= 9,1 Hz, 2H), 7.45 (s, 2H), 7.08 (d, *J*= 8.8 Hz, 2H), 3.45 (s, 4H), 1.62 (s, 6H).

**^13^C NMR** (150 MHz, DMSO-d_6_): *δ* (ppm) = 154.0, 153.4, 144.3, 143.3, 126.9, 125.3, 122.1, 113.8, 48.0, 24.9, 23.9.

**HR-MS** (ESI): calc. for C_17_H_21_N_4_O_2_S^+^ [M+H]^+^: 345.1380, found: 345.1389.

**R_t_** (LC-MS); MeCN/H_2_O/formic acid=10/90/0.1 to 95/5/0.1 over 7 min) = 4.694 min.

**λ_max_** (LC-MS) = 446 nm

## General procedure B for sulfonylurea installation

The following procedure to synthesize the sulfonylurea-containing azobenzenes (**4a-d**) was adapted from the literature and modified.^1^ The sulfonamide (**3a-d**) (35 mg, 0.1 mmol, 1.0 equiv.) and K_2_CO_3_ (21 mg, 0.15 mmol, 1.5 equiv.) were dissolved in acetone (15 mL) and stirred for 1 h at 70 °C before addition of cyclohexyl isocyanate (20 *µ*L, 0.16 mmol, 1.6 equiv.). The reaction mixture was stirred for an additional 5 h at 70 °C before cooling to 40 °C. The mixture was filtered, washed with acetone and dissolved in MeOH. All volatiles were removed *in vacuo*.

## (*E*)-*N*-(Cyclohexylcarbamoyl)-4-((4-(diethylamino)phenyl)diazenyl)- benzenesulfonamide (4a or JB253)


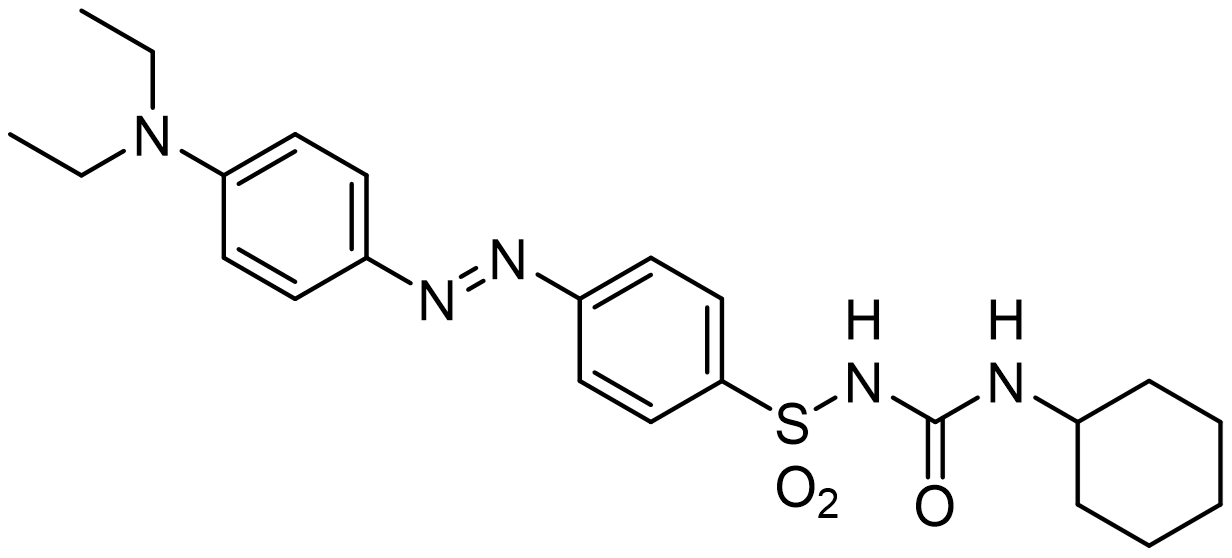


Column chromatography (1:2 EtOAc:cyclohexane) yielded 98% (45 mg) of the desired product as a red powder. For characterization, 20 mg was subjected to HPLC for purification.

**^1^H NMR** (600 MHz, DMSO-d_6_): *δ* (ppm) = 10.42 (s, 1H), 8.01 (d, *J*= 8.7 Hz, 2H), 7,89 (d, *J*= 8.7 Hz, 2H), 7.82 (d, *J*= 9.2 Hz, 2H), 6.84 (d, *J*= 9.3 Hz, 2H), 6.41 (d, *J*= 7.6 Hz, 1H), 3.49 (q, *J*= 7.1 Hz, 4H), 1.67-1.65 (m, 2H), 1.60-1.57 (m, 2H), 1.49-1.46 (m, 1H), 1.22-1.10 (m, 11H).

**^13^C NMR** (150 MHz, DMSO-d_6_): *δ* (ppm) = 155.1, 150.9, 150.4, 142.2, 139.6, 128.6, 125.9, 121.8, 111.2, 48.1, 44.2, 32.2, 24.9, 24.2, 12.5.

**HR-MS** (ESI): calc. for C_23_H_32_N_5_O_3_S^+^ [M+H]^+^: 458.2220, found: 458.2231.

**R_t_** (LC-MS); MeCN/H_2_O/formic acid=10/90/0.1 to 95/5/0.1 over 7 min) = 5.583 min.

**λ_max_** (LC-MS) = 466 nm; **λ_max_** (UV/Vis) = 472 nm

## (*E*)-*N*-(Cyclohexylcarbamoyl)-4-((4-(azetidin-1-yl)phenyl)diazenyl)- benzenesulfonamide (4b or JB1793)


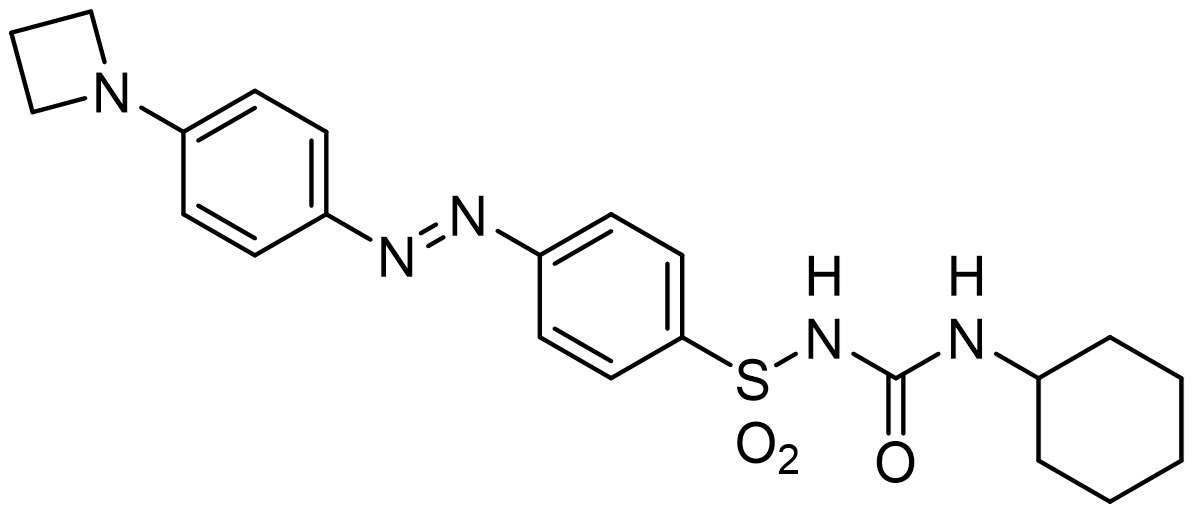


The crude product was purified via HPLC yielding 54% (24 mg) of the desired product as a red powder.

**^1^H NMR** (600 MHz, DMSO-d_6_): *δ* (ppm) = 10.42 (s. 1H), 8.02 (d, *J*= 8.6 Hz, 2H), 7.90 (d, *J*= 8.6 Hz, 2H), 7.82 (d, *J*= 8.8 Hz, 2H), 6.51 (d, *J*= 8.9 Hz, 2H), 6.40 (d, *J*= 7.5 Hz, 1H), 4.03 (t, *J*= 7.4 Hz, 4H) 3.29 (m, 1H), 2.39 (m, 2H), 1.66 (m, 2H), 1.58 (m, 2H), 1.48 (m, 1H), 1.21 (m, 2H), 1.11 (m, 3H).

**^13^C NMR** (150 MHz, DMSO-d_6_): *δ* (ppm) = 154.9, 153.9, 150.4, 143.1, 139.9, 128.6, 125.5, 121.9, 110.3, 51.3, 48.1, 32.2, 24.9, 24.2, 15.8.

**HR-MS** (ESI): calc. for C_22_H_28_N_5_O_3_S^+^ [M+H]^+^: 442.1907, found: 442.1920.

**R_t_** (LC-MS); MeCN/H_2_O/formic acid=10/90/0.1 to 95/5/0.1 over 7 min) = 5.351 min.

**λ_max_** (LC-MS) = 454 nm; **λ_max_** (UV/Vis) = 454 nm

## €-*N*-(Cyclohexylcarbamoyl)-4-((iazinylnedin-1-yl)pheniazinylnyl)- benzenesulfonamide (4c)


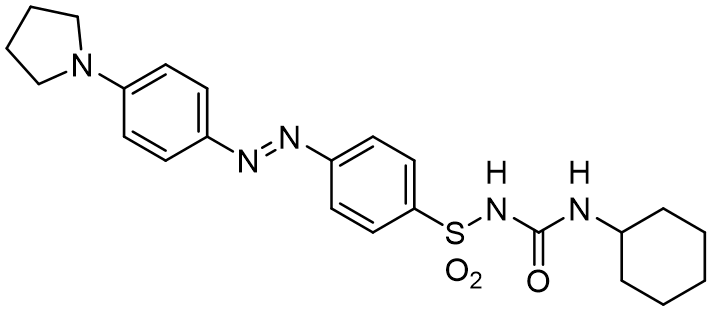


Column chromatography (1:1 EtOAc:cyclohexane) yielded 43% (20 mg) of the desired product as a red powder. For characterization, 20 mg was subjected to HPLC for purification.

**^1^H NMR** (600 MHz, DMSO-d_6_): *δ* (ppm) = 10.41 (s, 1H), 8.00 (d, *J*= 8.7 Hz, 2H), 7.89 (d, *J*= 8.7 Hz, 2H), 7.83 (d, *J*= 9.1 Hz, 2H), 6.71 (d, *J*= 9.1 Hz, 2H), 6.40 (d, *J*= 7.7 Hz, 1H), 3.40, (t, *J*= 6.6 Hz, 4H), 3.28 (m, 1H), 2.00 (m, 4H), 1.66 (m, 2H), 1.58 (m, 2H), 1.47 (m, 1H), 1.20 (m, 2H), 1.11 (m, 3H).

**^13^C NMR** (150 MHz, DMSO-d_6_): *δ* (ppm) = 155.0, 150.7, 150.4, 142.5, 139.6, 128.6, 125.8, 121.8, 111.8, 48.1, 47.6, 32.2, 24.9, 24.2.

**HR-MS** (ESI): calc. for C_23_H_30_N_5_O_3_S^+^ [M+H]^+^: 456.2064, found: 456.2079.

**R_t_** (LC-MS); MeCN/H_2_O/formic acid=10/90/0.1 to 95/5/0.1 over 7 min) = 5.578 min.

**λ_max_** (LC-MS) = 470 nm; **λ_max_** (UV/Vis) = 475 nm

## (*E*)-*N*-(Cyclohexylcarbamoyl)-4-((4-(piperidin-1-yl)phenyl)diazenyl)- benzenesulfonamide (4d or JB1794)


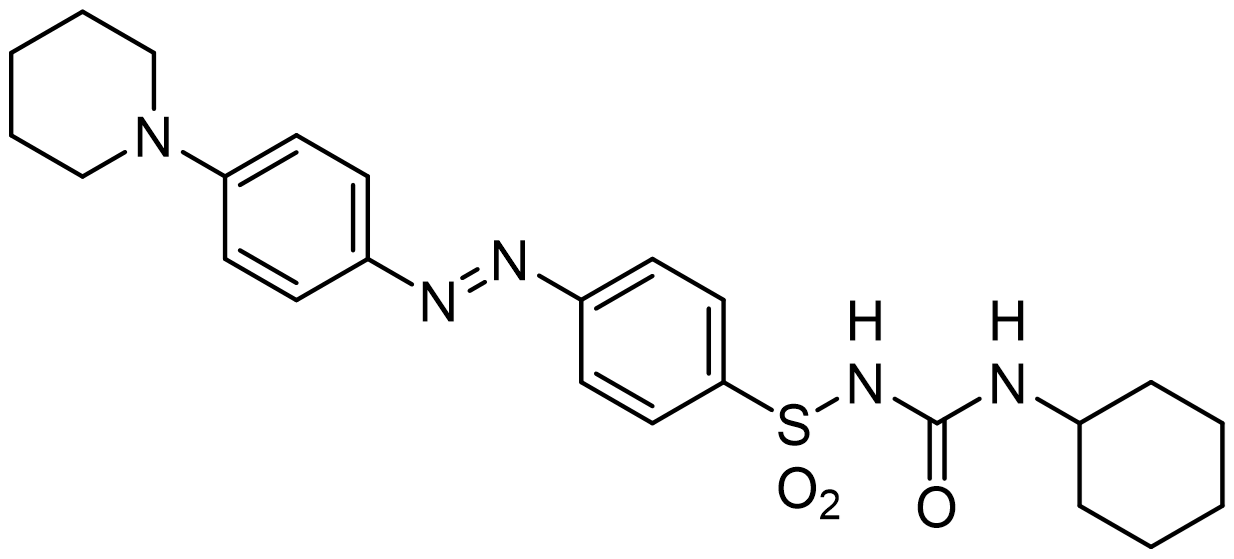


Column chromatography (1:1 EtOAc:cyclohexane) yielded 61% (29 mg) of the desired product as a red powder. For characterization, 20 mg was subjected to HPLC for purification.

**^1^H NMR** (600 MHz, DMSO-d_6_): *δ* (ppm) = 10.43 (s, 1H), 8.03 (d, *J*= 8.7 Hz, 2H), 7.91 (d, *J*= 8.7 Hz, 2H), 7.81 (d, *J*= 9.2 Hz, 2H), 7.07 (d, *J*= 9.3 Hz, 2H), 6.40 (d, *J*= 7.6 Hz, 1H), 3.45 (t, *J*= 4.7 Hz, 4H), 3.29 (m, 1H), 1.66 (m, 2H), 1.59 (m, 8H), 1.48 (m, 1H), 1.21 (m, 2H), 1.12 (m, 3H).

**^13^C NMR** (150 MHz, DMSO-d_6_): *δ* (ppm) = 154.9, 153.5, 150.4, 143.2, 140.0, 128.6, 125.4, 122.0, 113.6, 48.1, 47.9, 32.2, 24.9, 24.2, 23.9.

**HR-MS** (ESI): calc. for C_24_H_32_N_5_O_3_S^+^ [M+H]^+^: 470.2220, found: 470.2226.

**R_t_** (LC-MS); MeCN/H_2_O/formic acid=10/90/0.1 to 95/5/0.1 over 7 min) = 5.734 min.

**λ_max_** (LC-MS) = 450 nm; **λ_max_** (UV/Vis) = 459 nm

# NMR spectra

## 1-Phenylazetidine


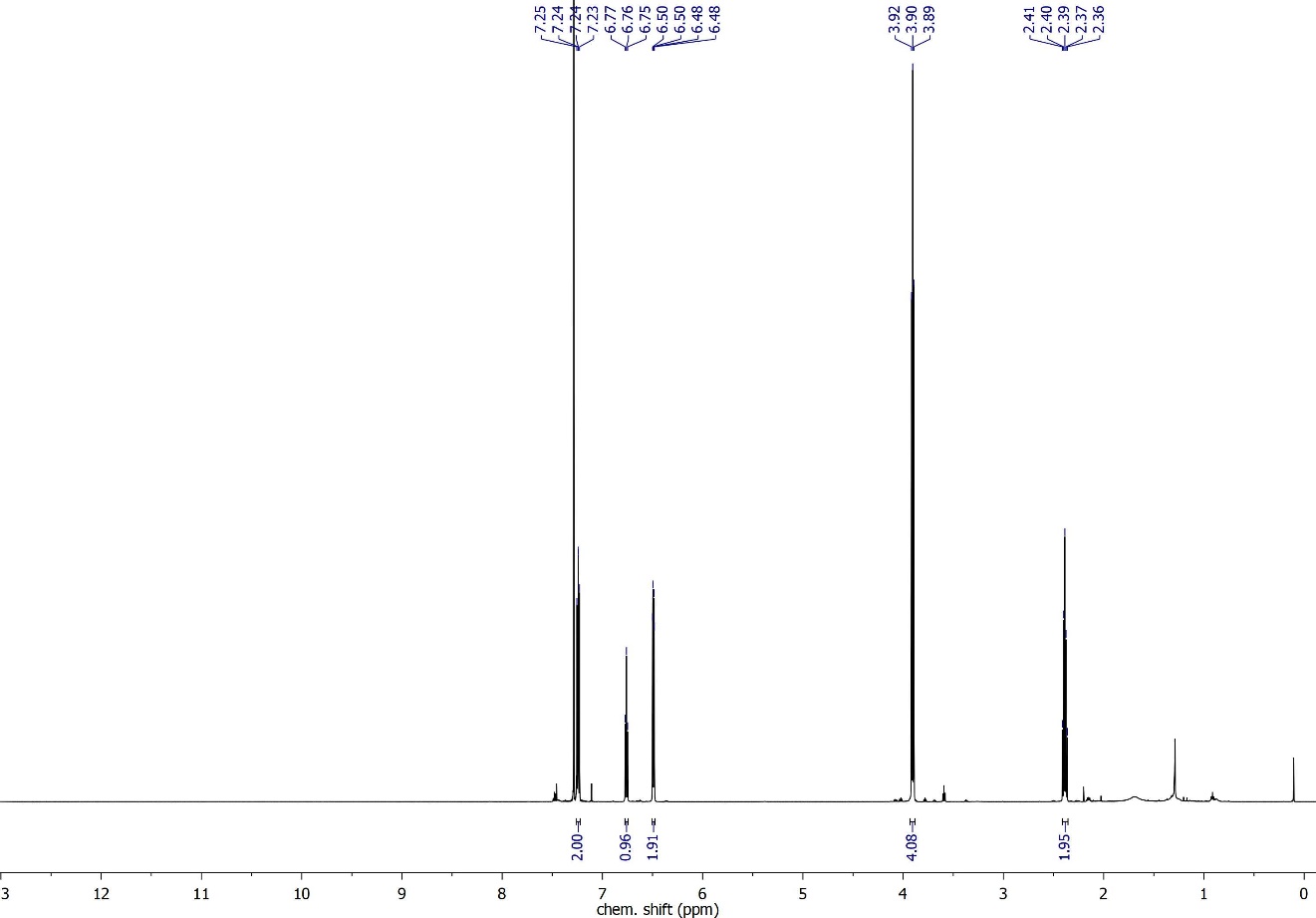

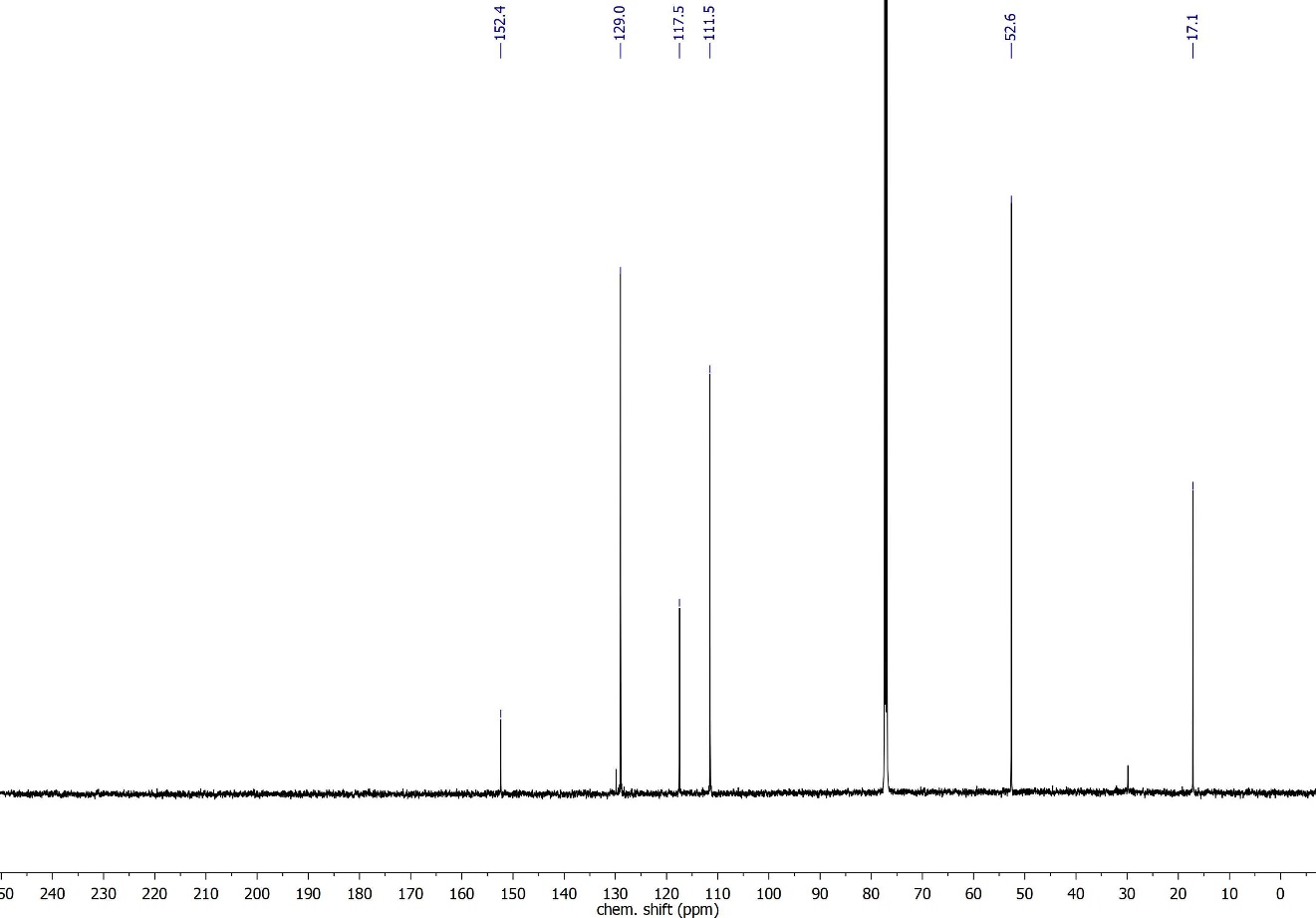


## (*E*)-4-((4-(Diethylamino)phenyl)diazenyl)benzenesulfonamide (3a)


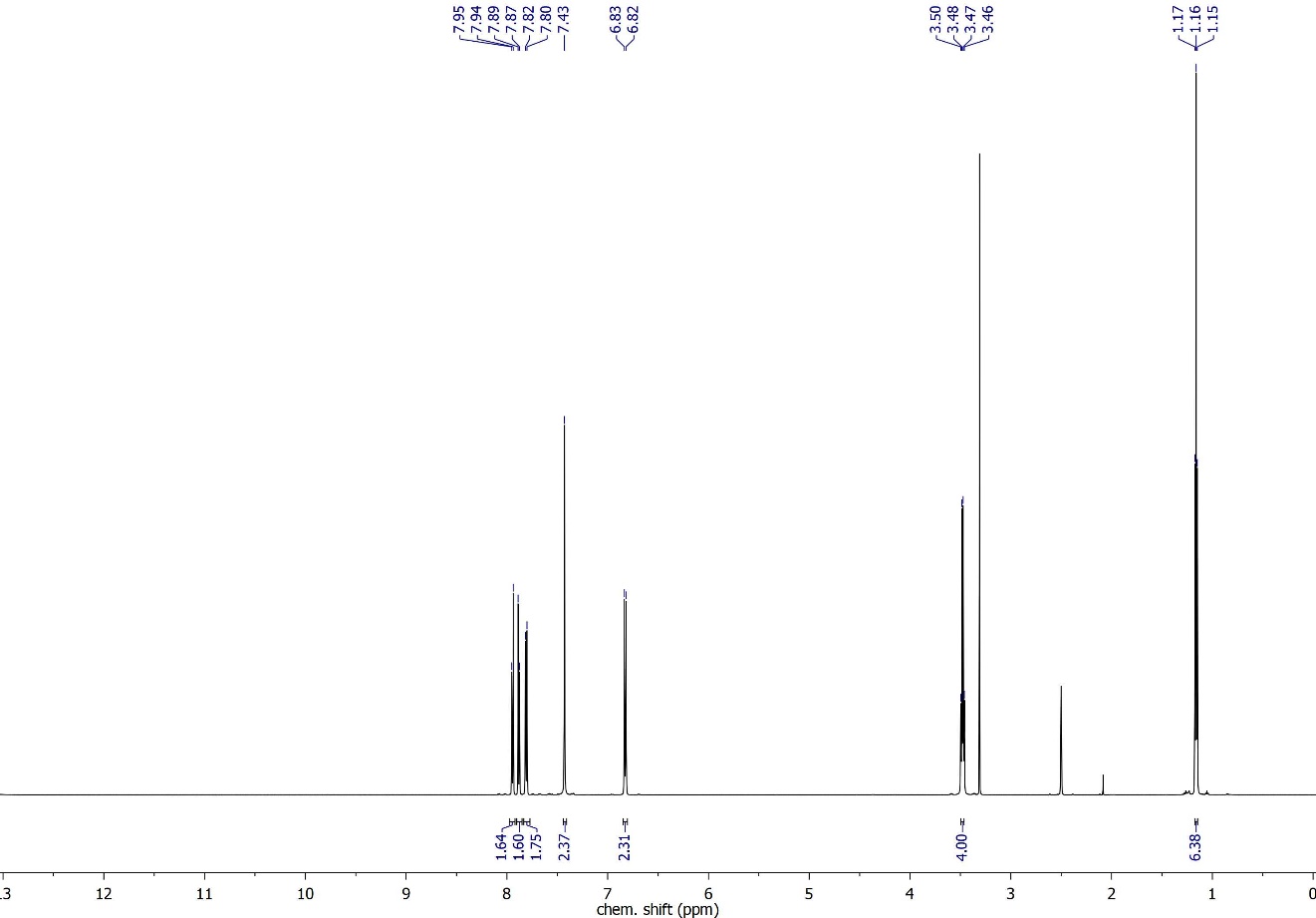

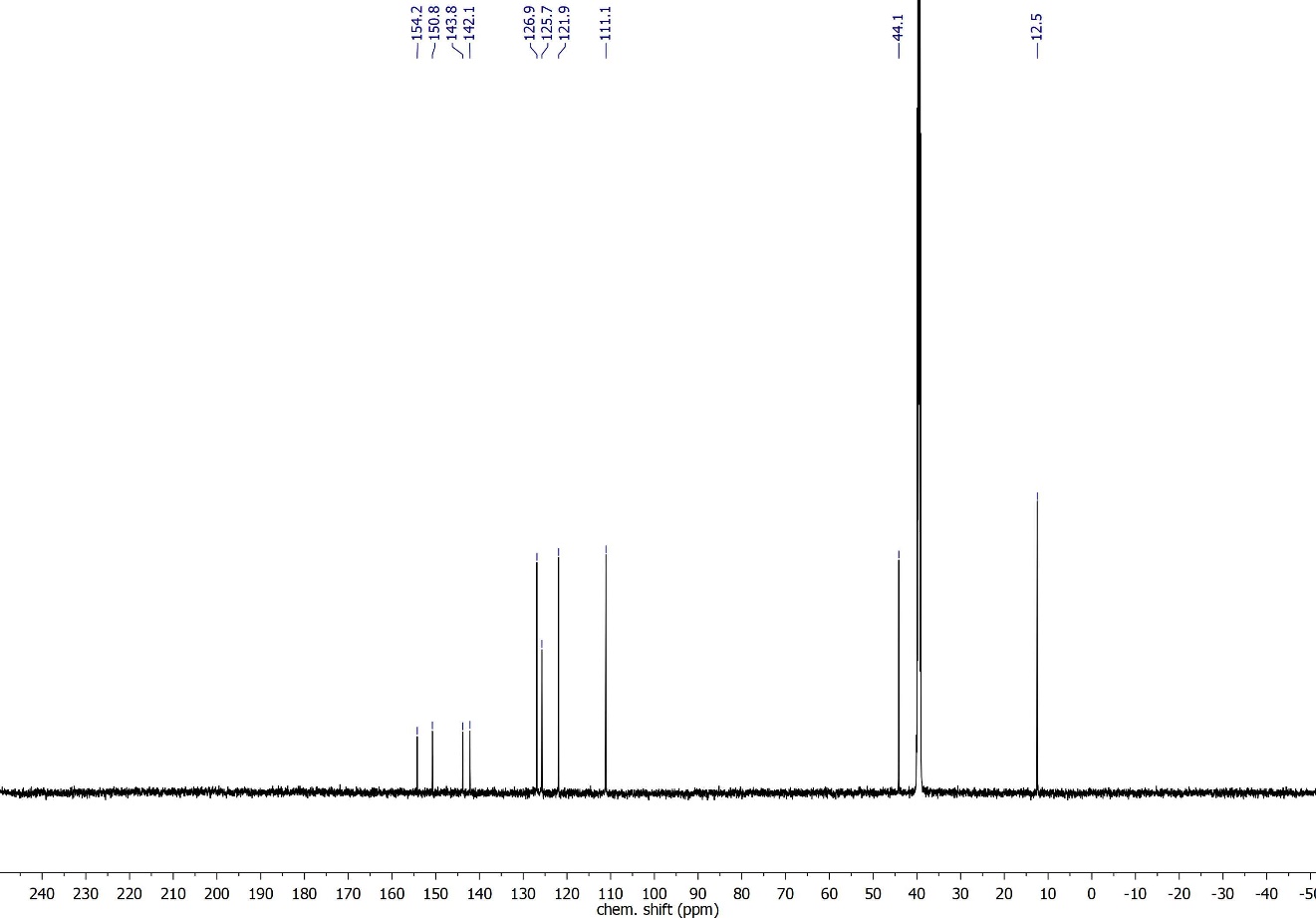


## (*E*)-4-((4-(Azetidin-1-yl)phenyl)diazenyl)benzenesulfonamide (3b)

**
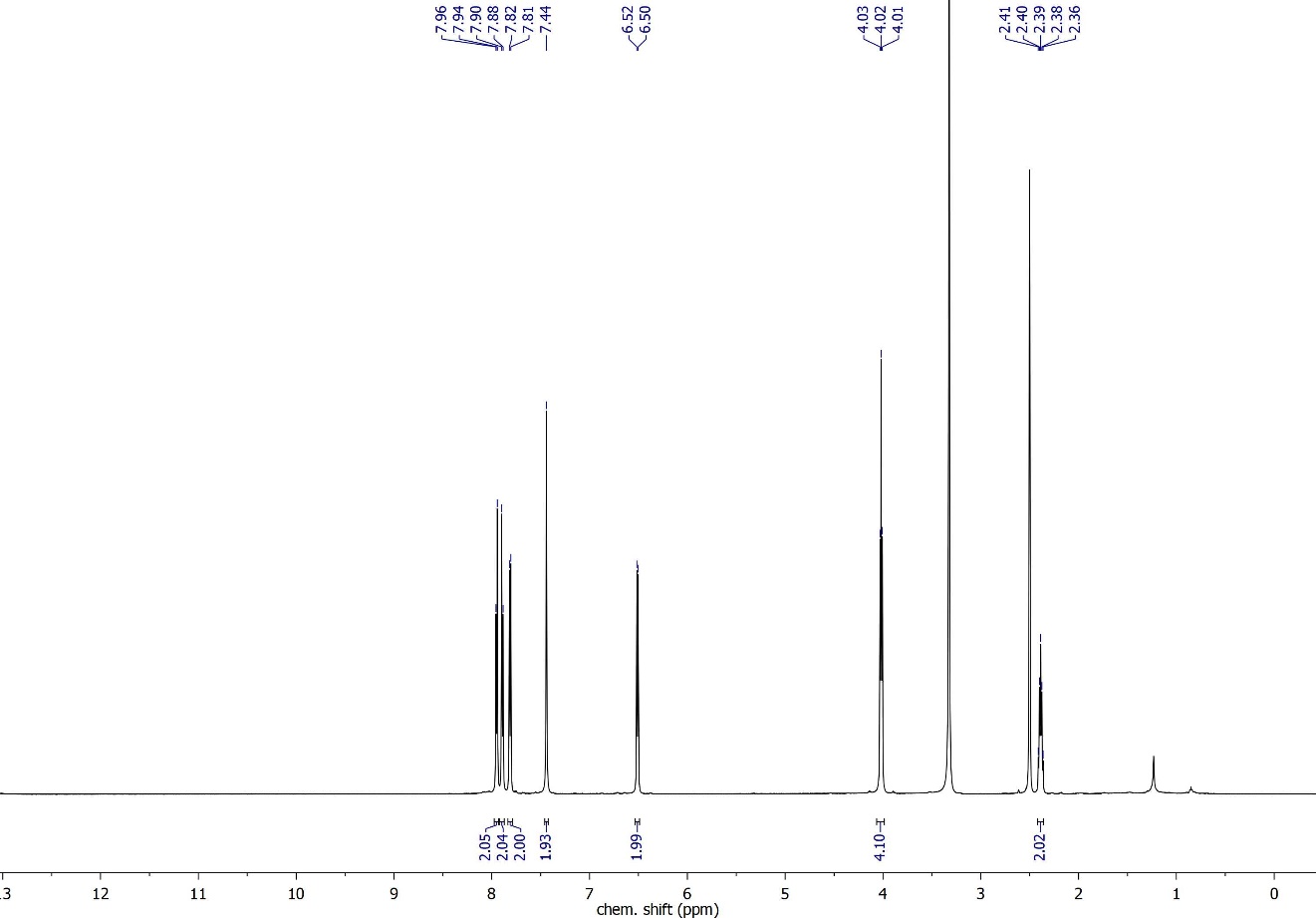

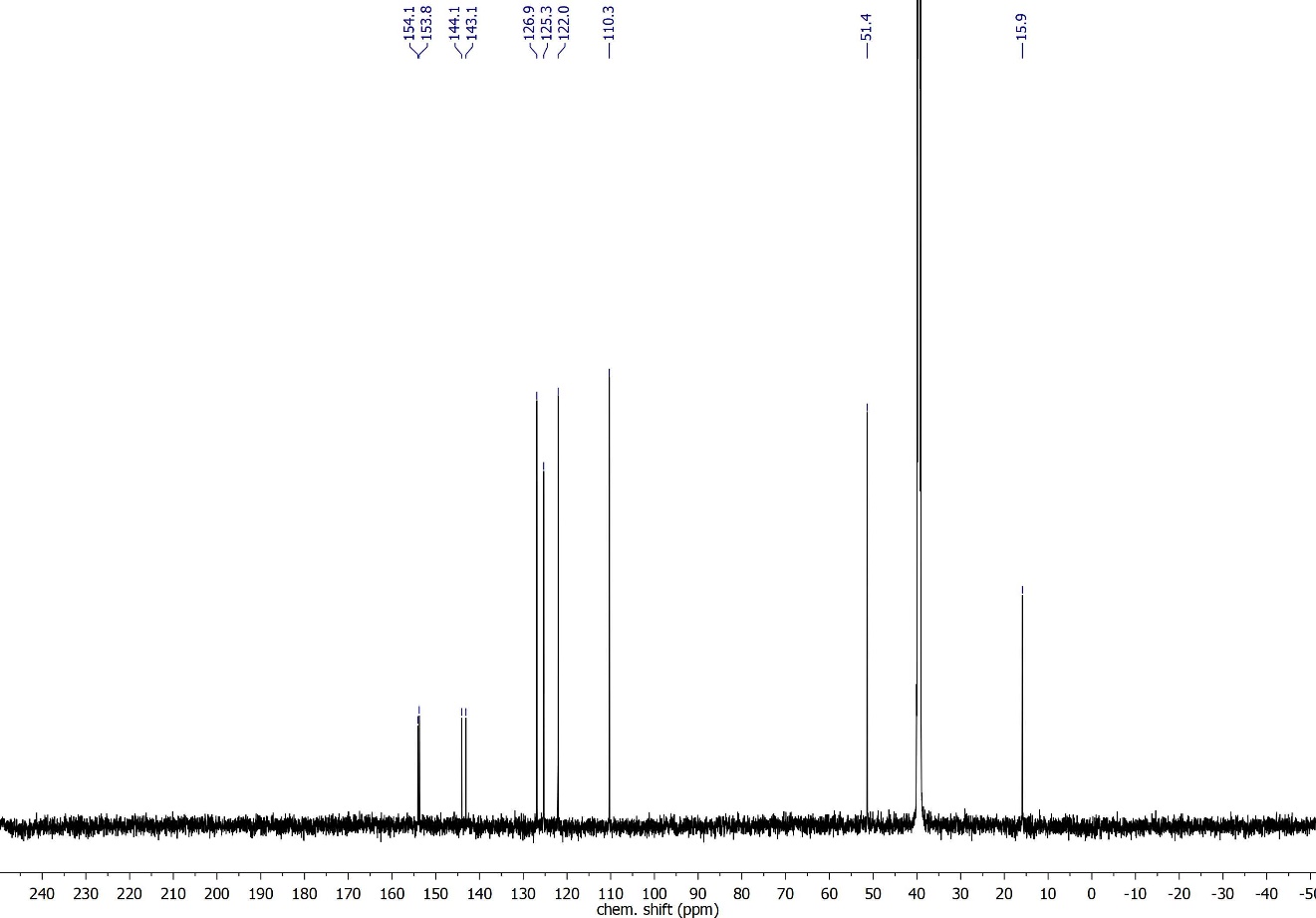
**

## (*E*)-4-((4-(Pyrrolidin-1-yl)phenyl)diazenyl)benzenesulfonamide (3c)

**
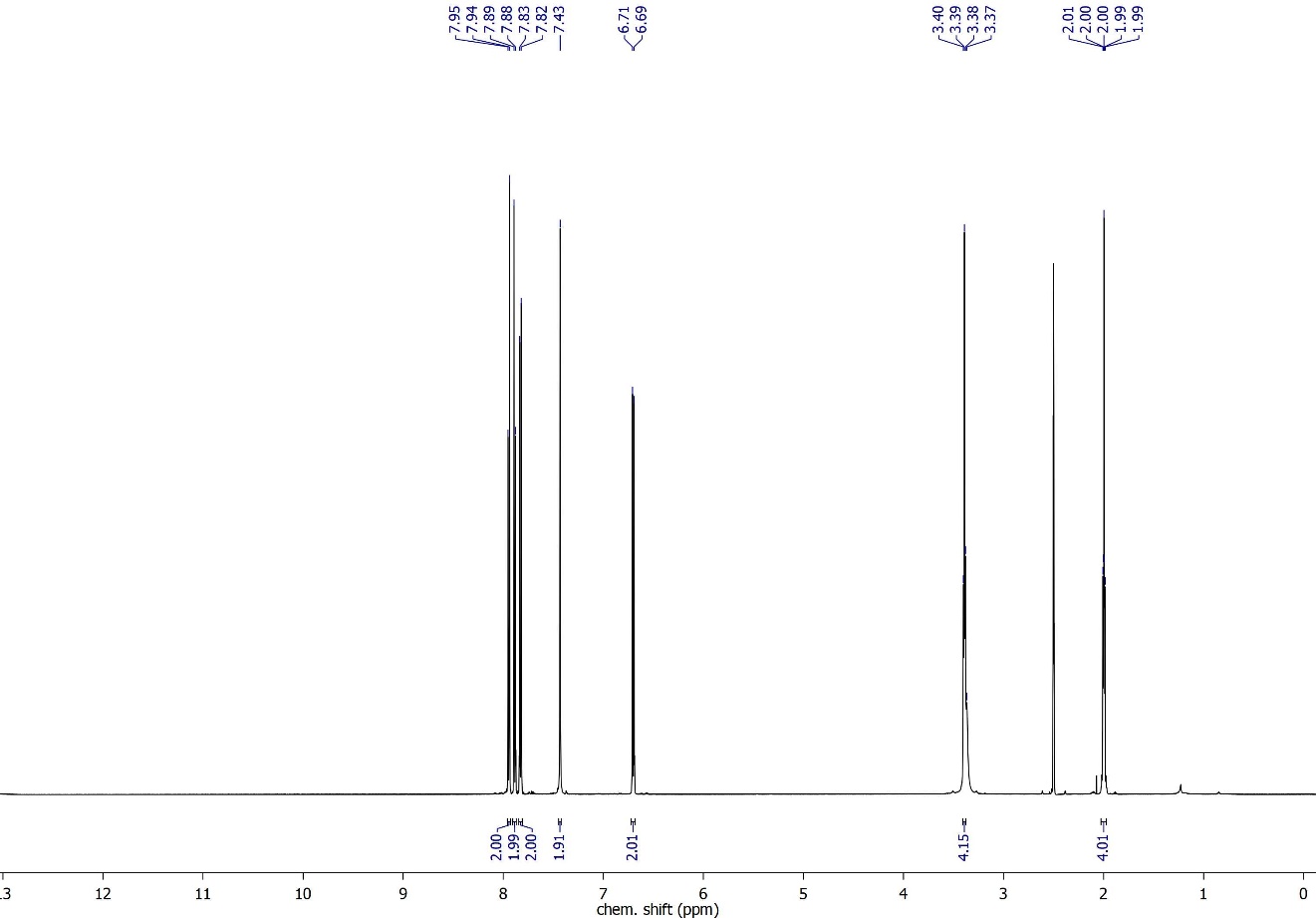

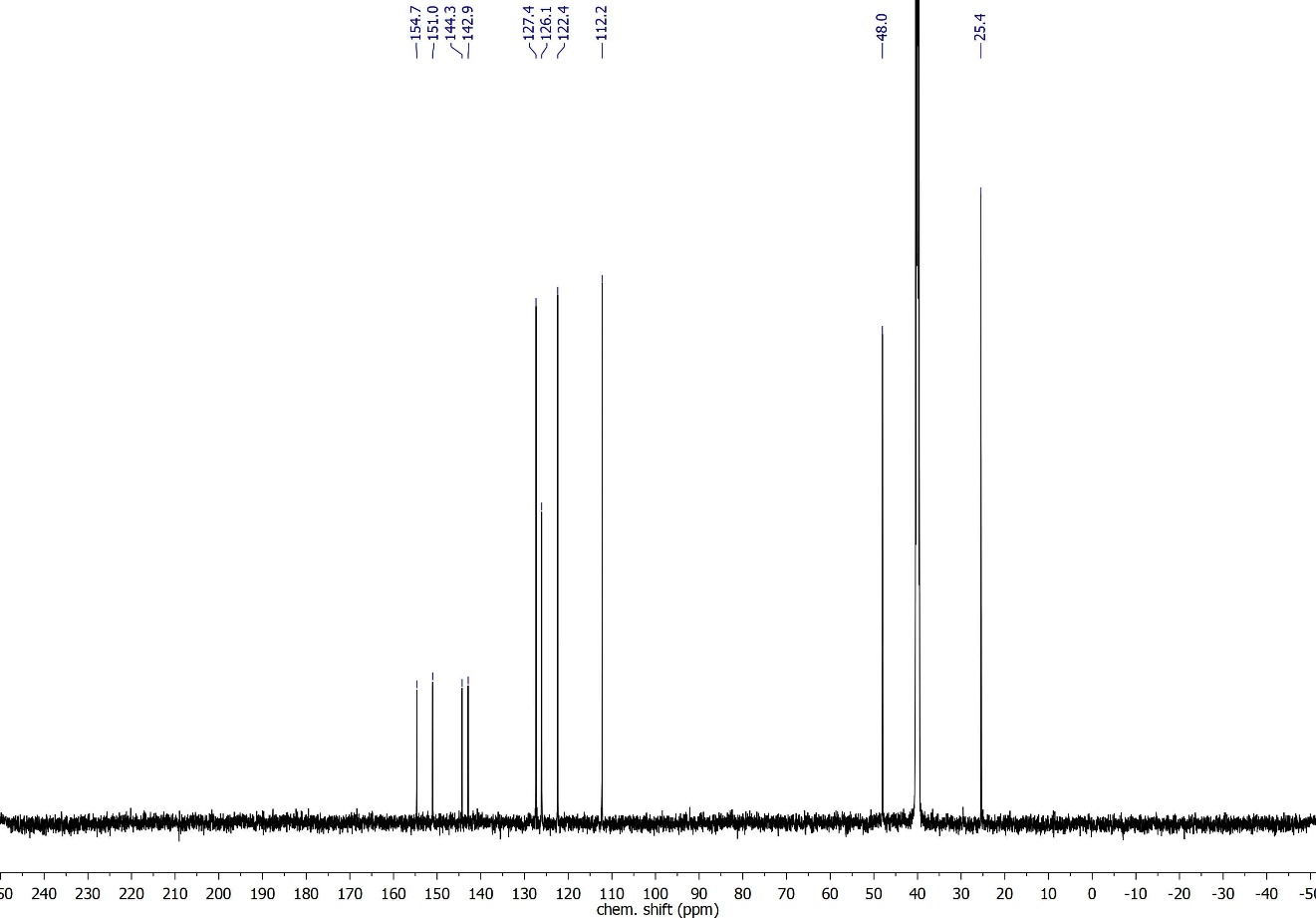
**

## (*E*)-4-((4-(Piperidin-1-yl)phenyl)diazenyl)benzenesulfonamide (3d)

**
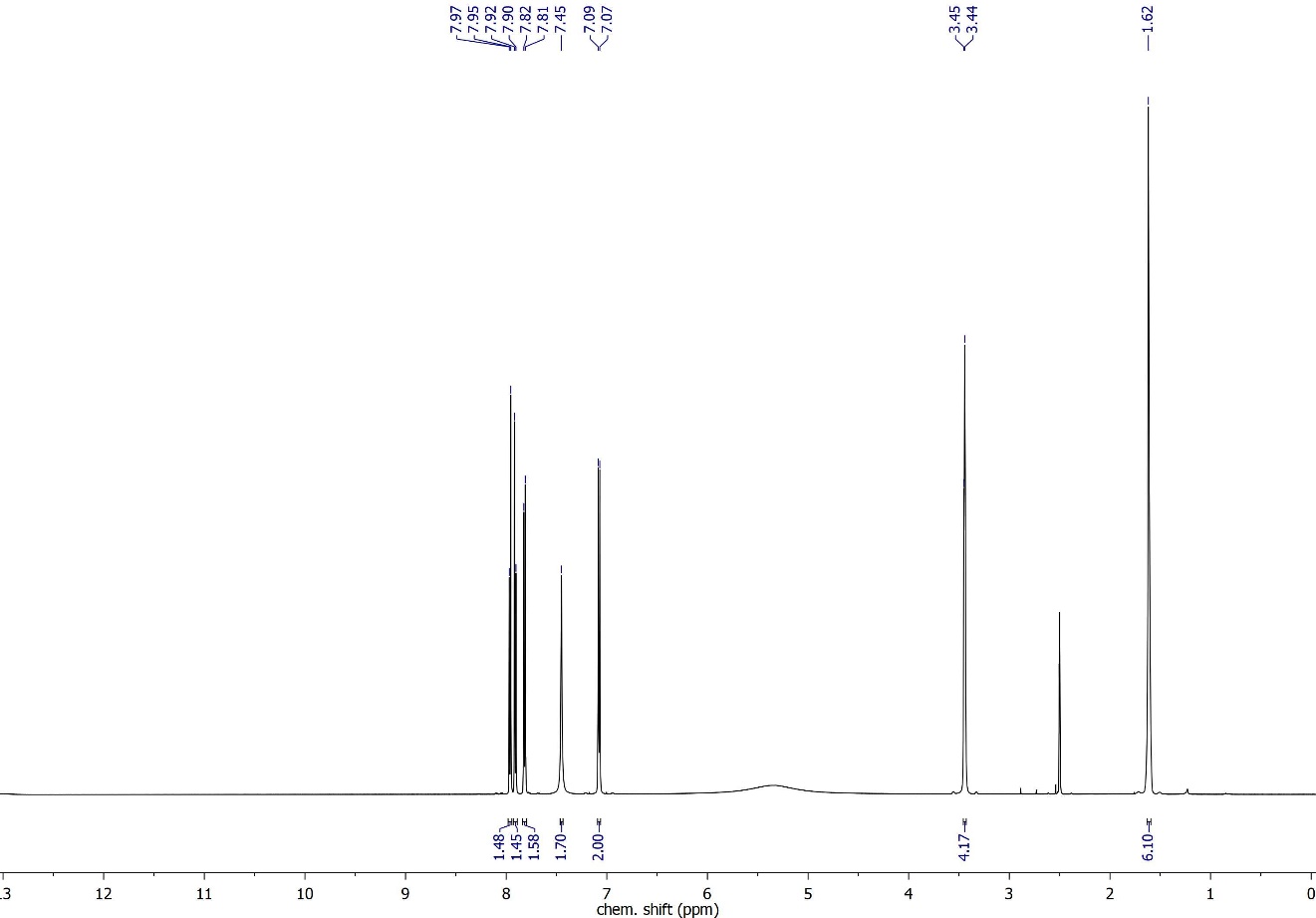

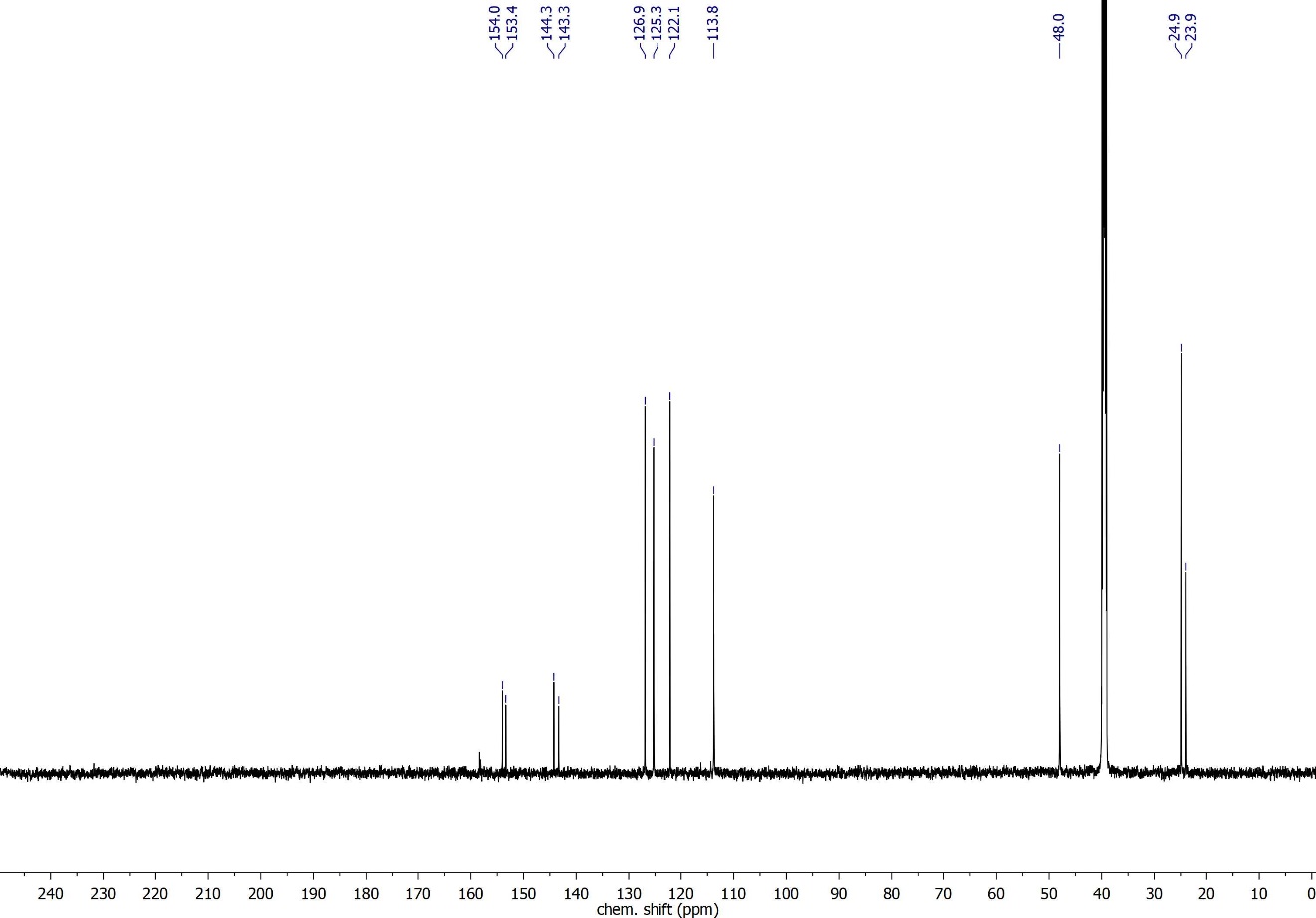
**

## (*E*)-*N*-(Cyclohexylcarbamoyl)-4-((4-(diethylamino)phenyl)diazenyl)- benzenesulfonamide (4a or JB253)

**
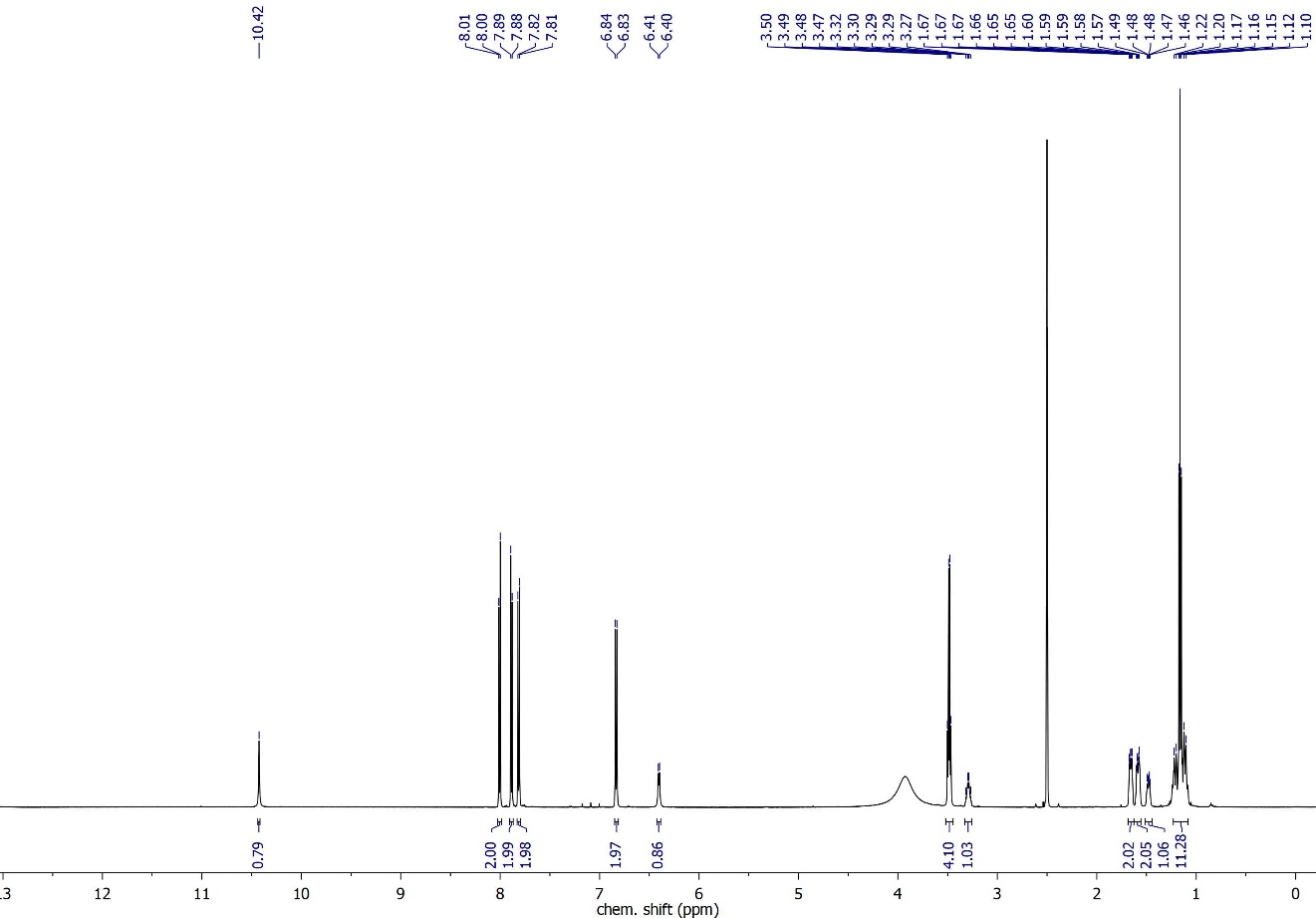

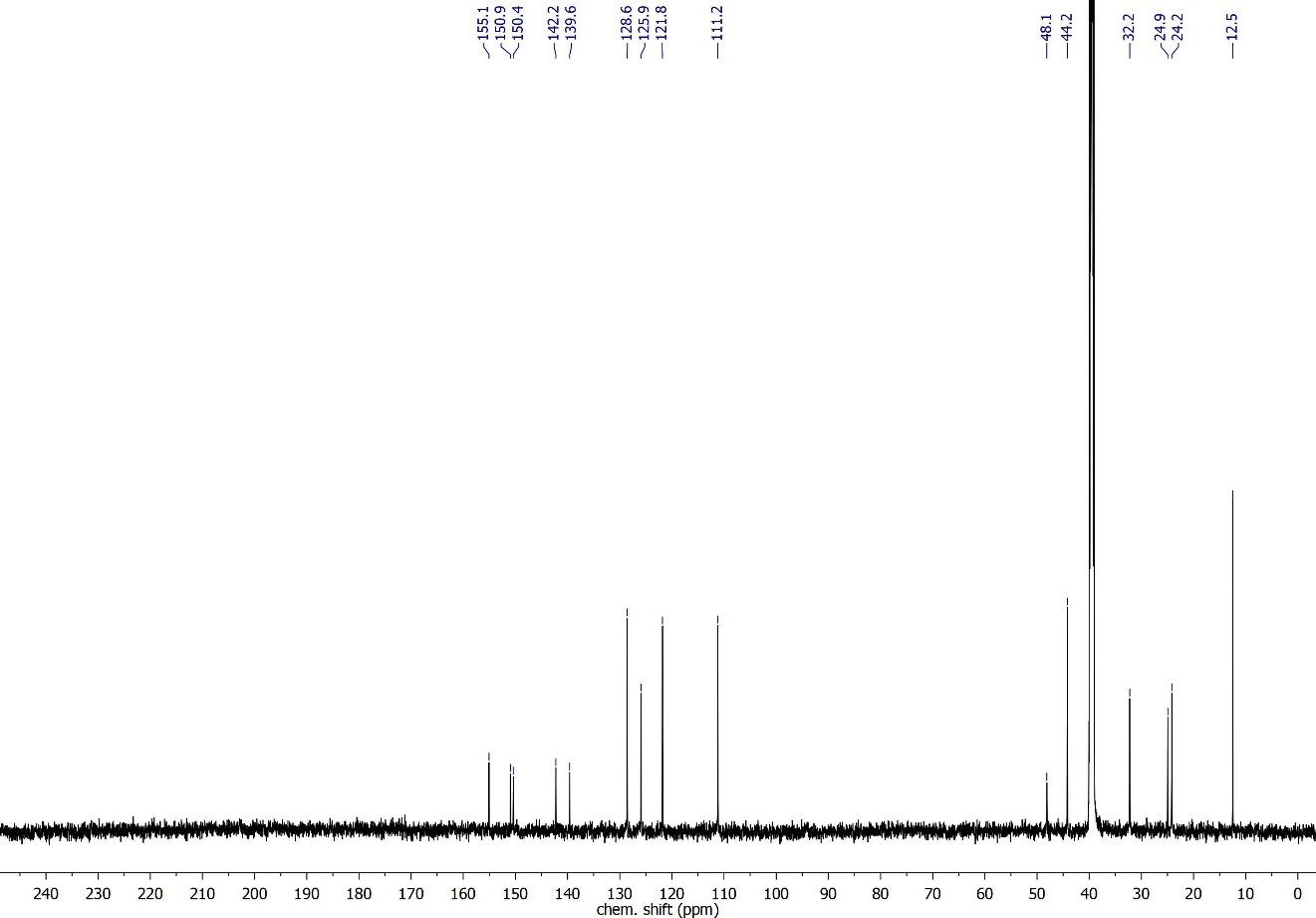
**

## (*E*)-*N*-(Cyclohexylcarbamoyl)-4-((4-(azetidin-1-yl)phenyl)diazenyl)- benzenesulfonamide (4b or JB1793)

**
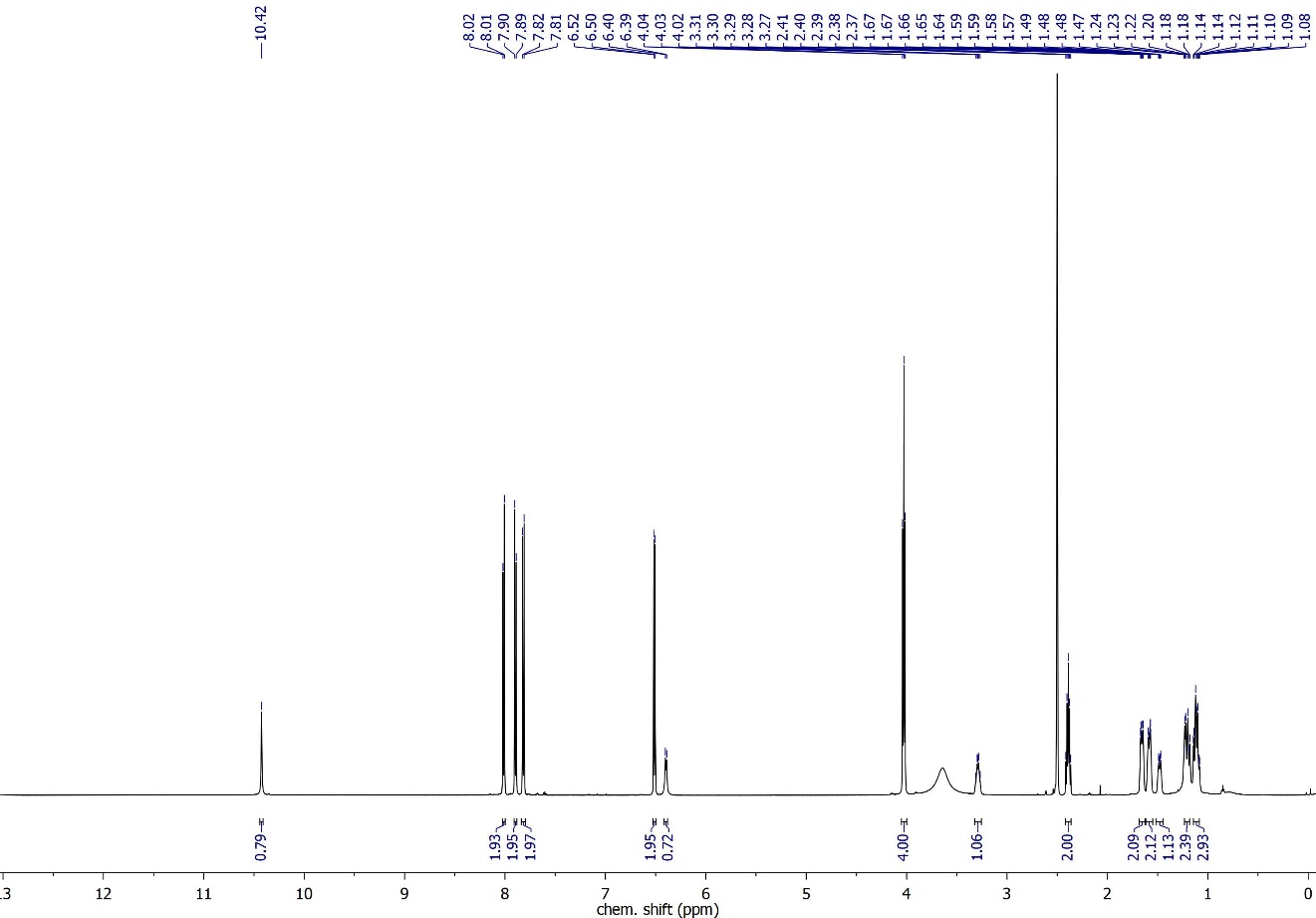
**

**
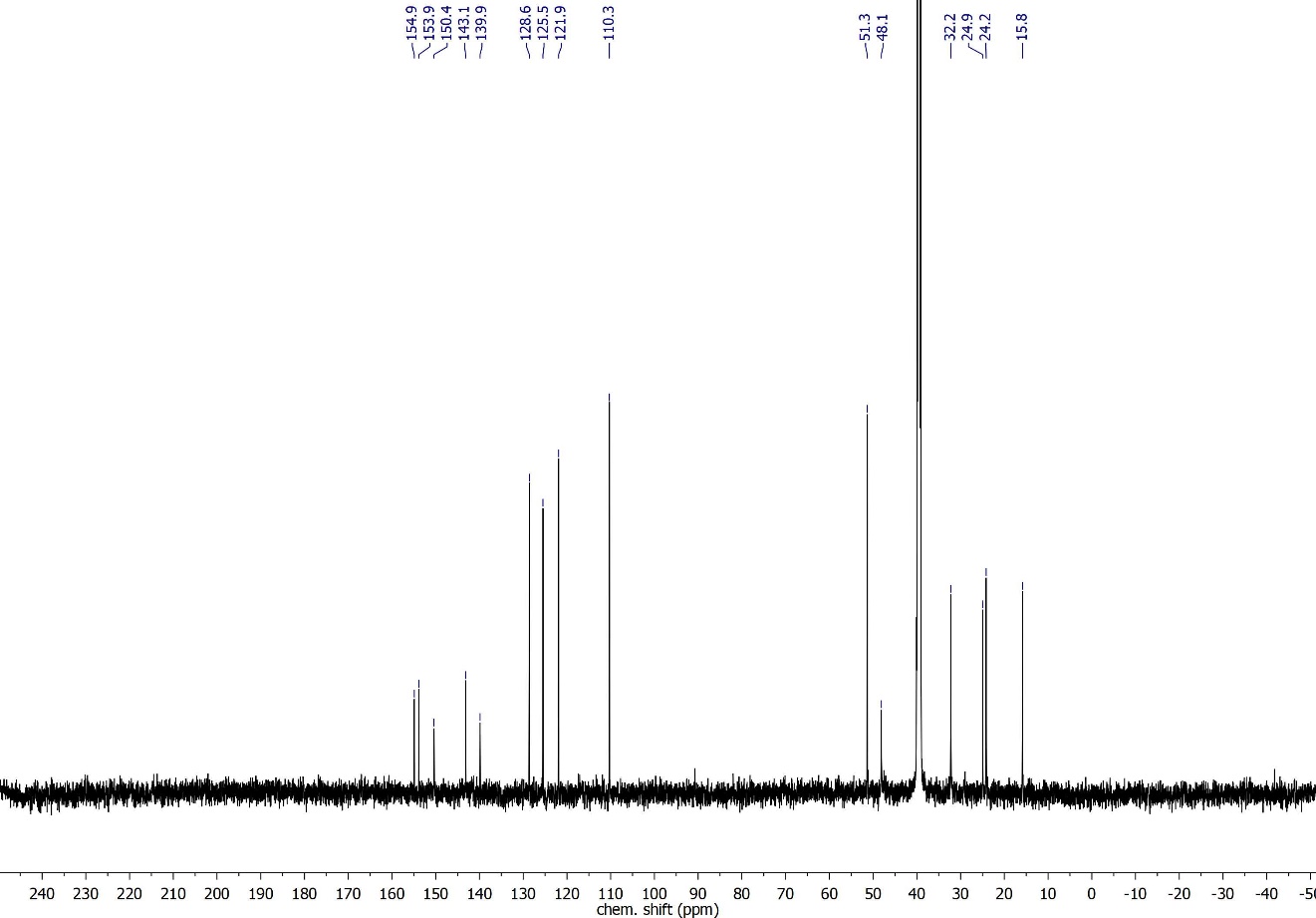
**

## (*E*)-*N*-(Cyclohexylcarbamoyl)-4-((4-(pyrrolidin-1-yl)phenyl)diazenyl)- benzenesulfonamide (4c or JB1794)

**
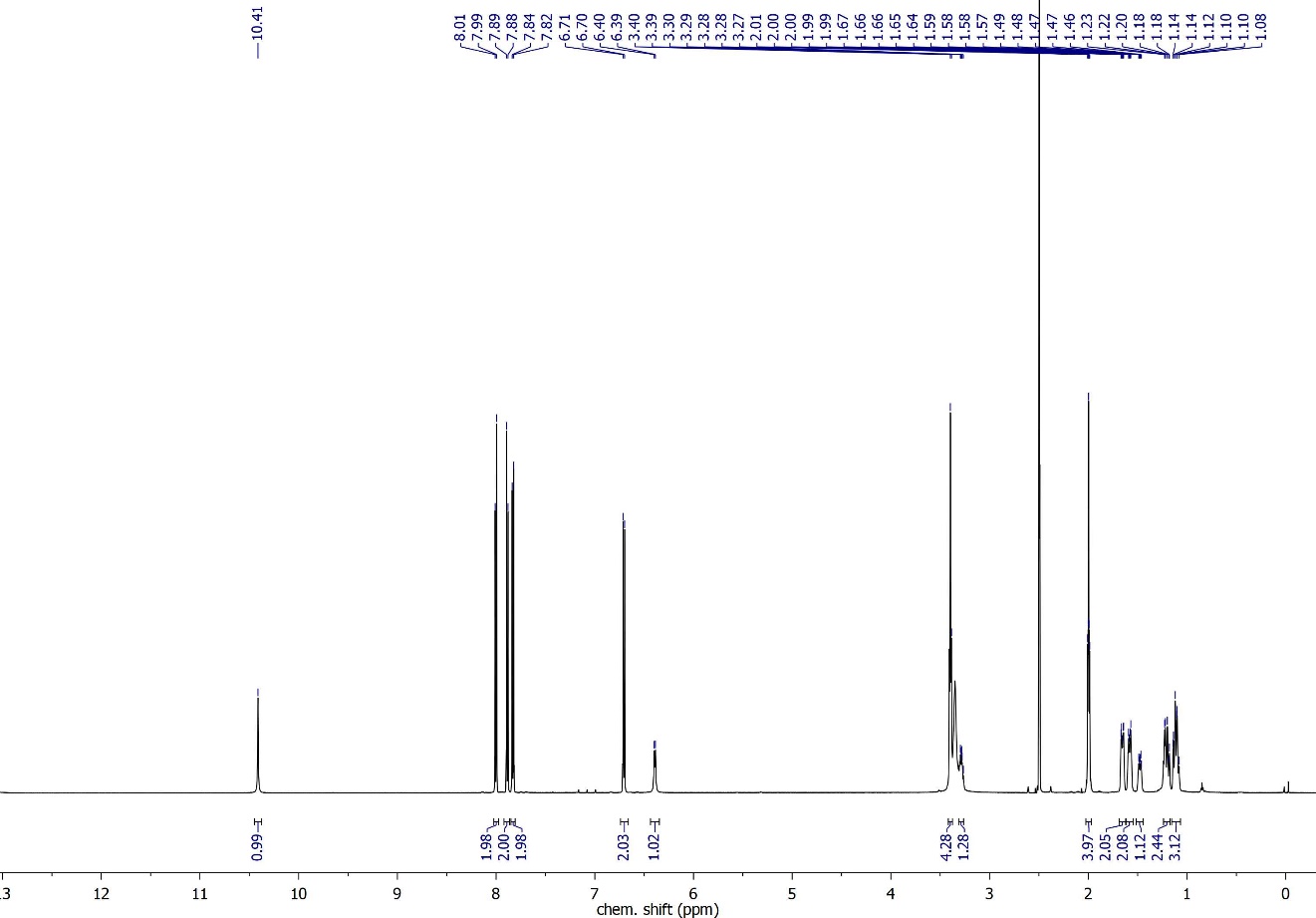

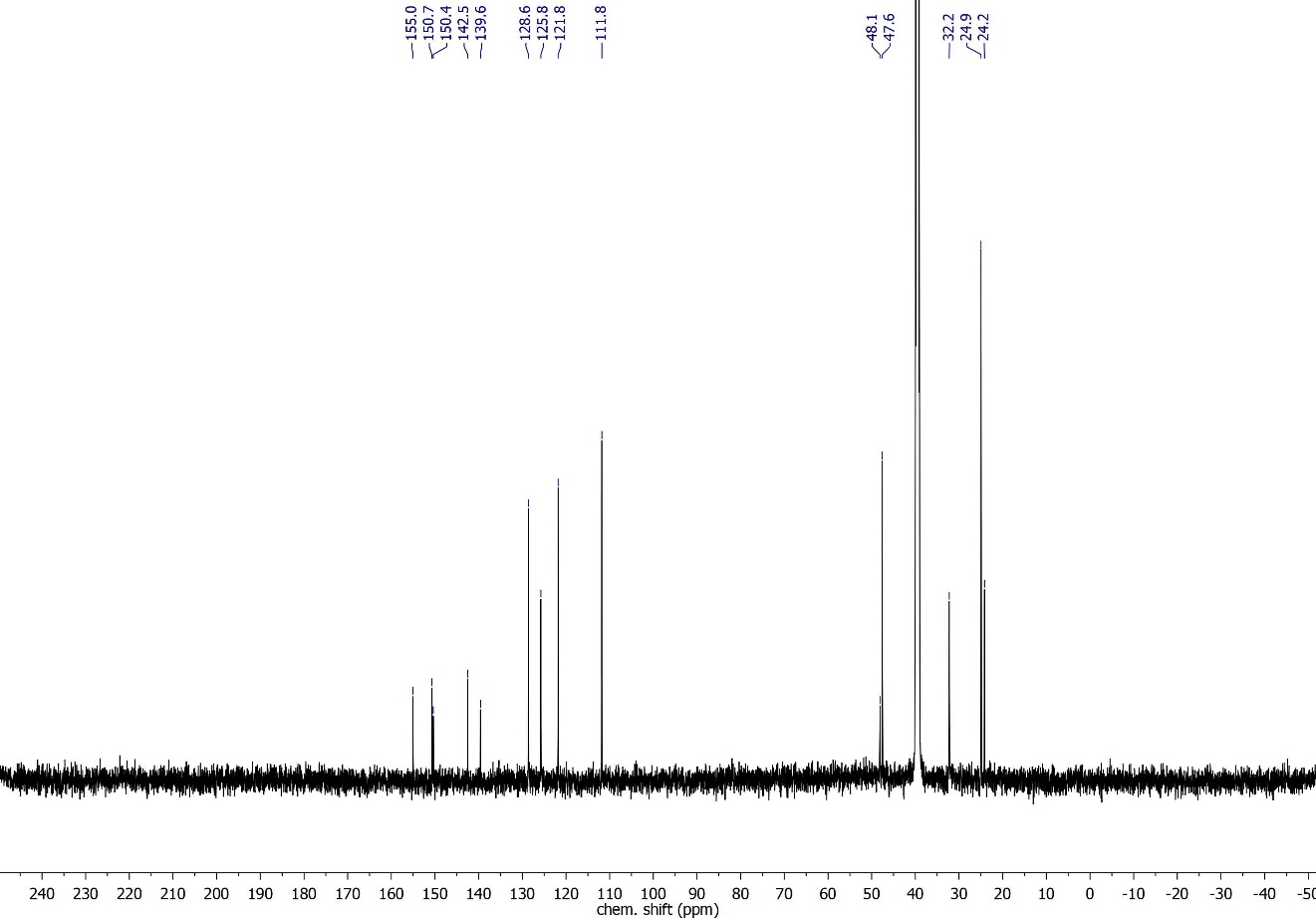
**

**
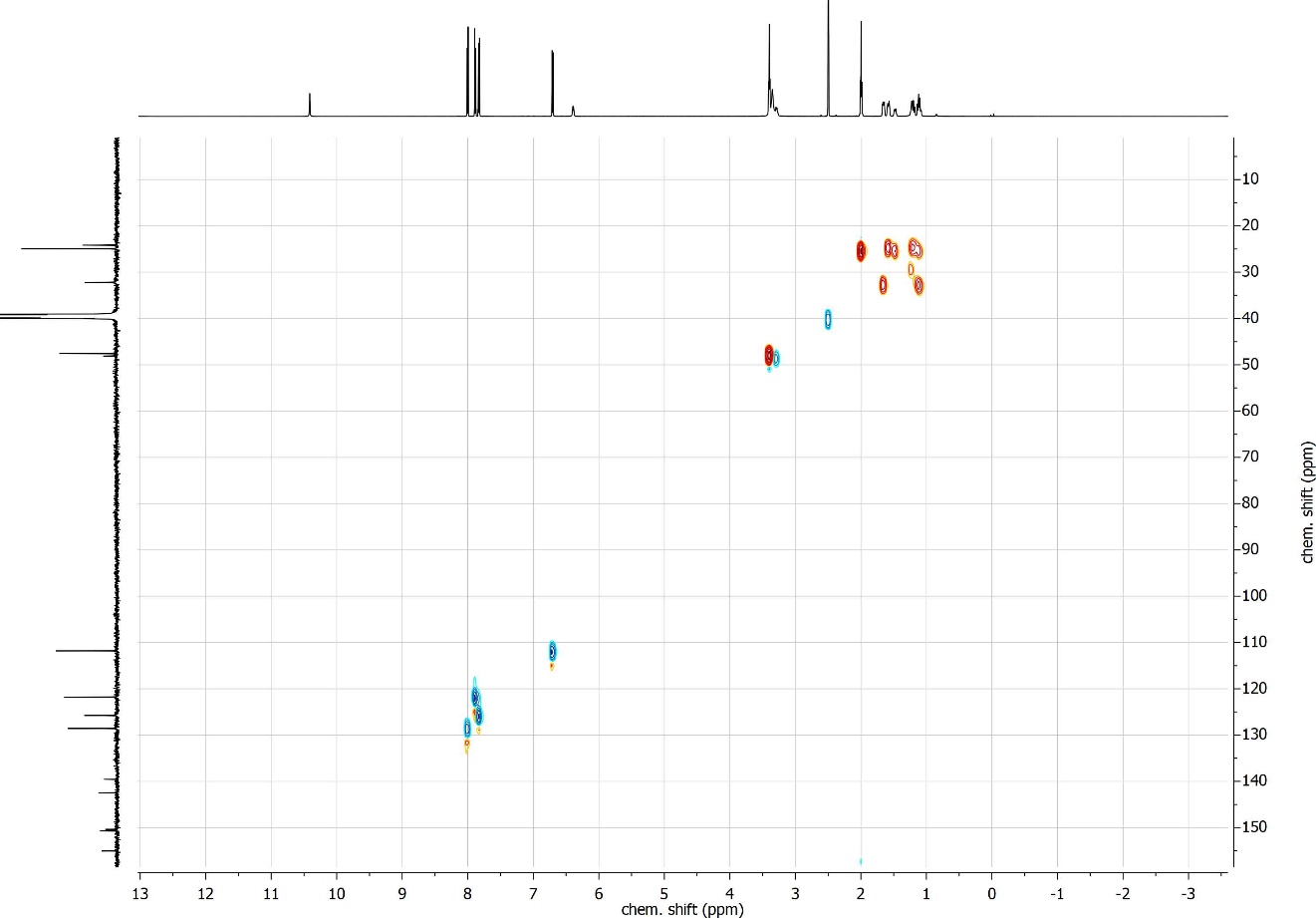
**

## (*E*)-*N*-(Cyclohexylcarbamoyl)-4-((4-(piperidin-1-yl)phenyl)diazenyl)- benzenesulfonamide (4d or JB1795)

**
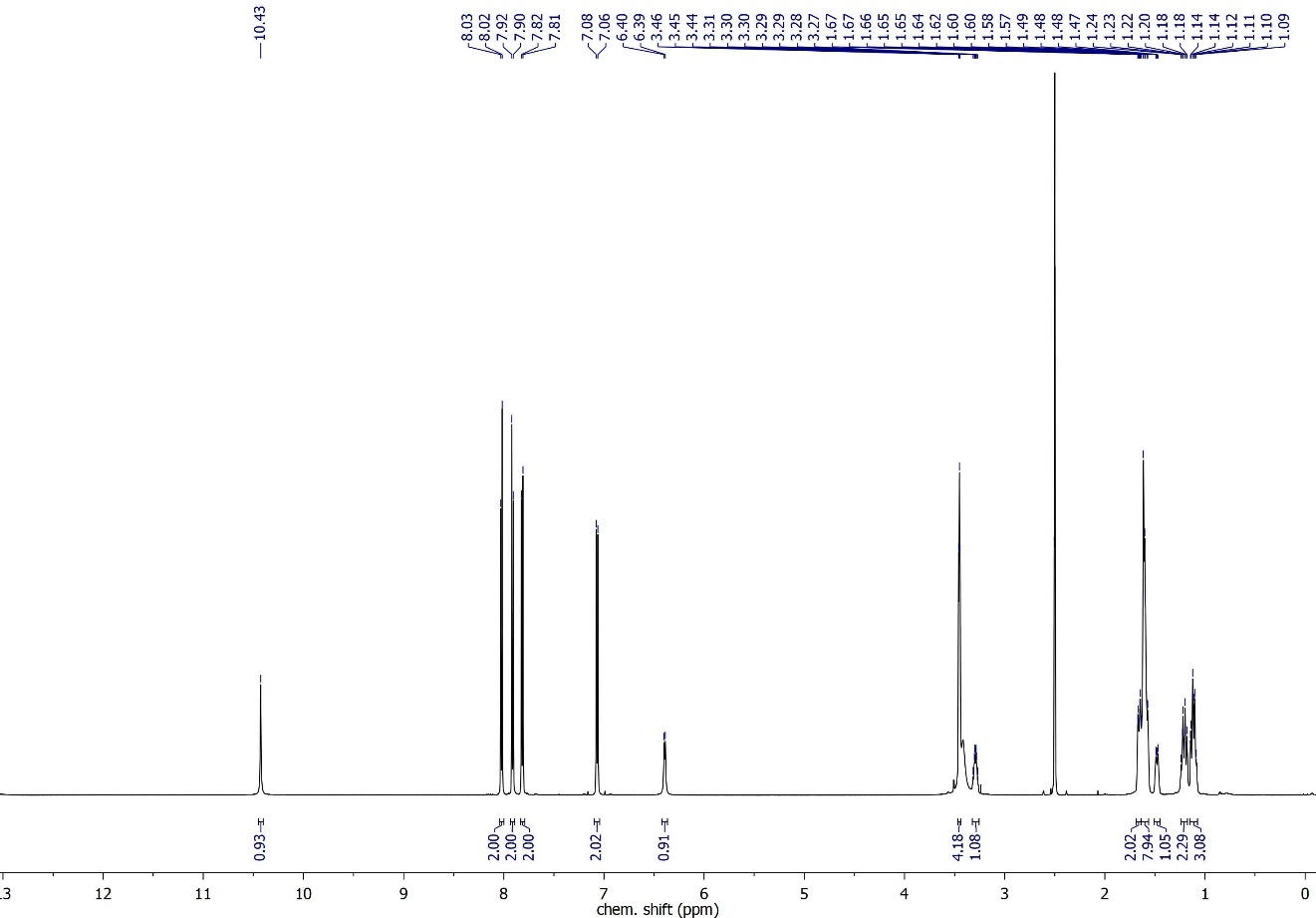

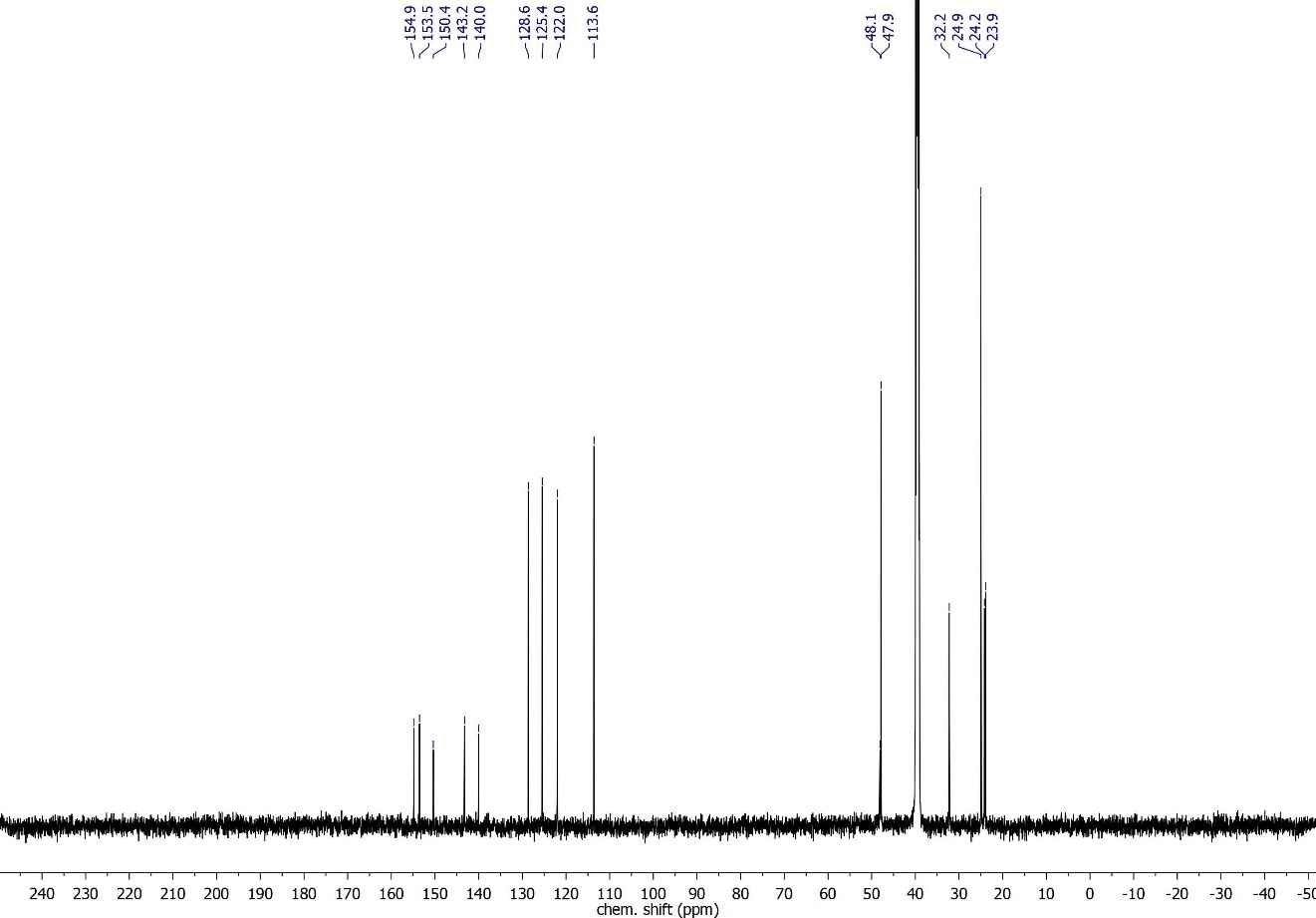
**

**
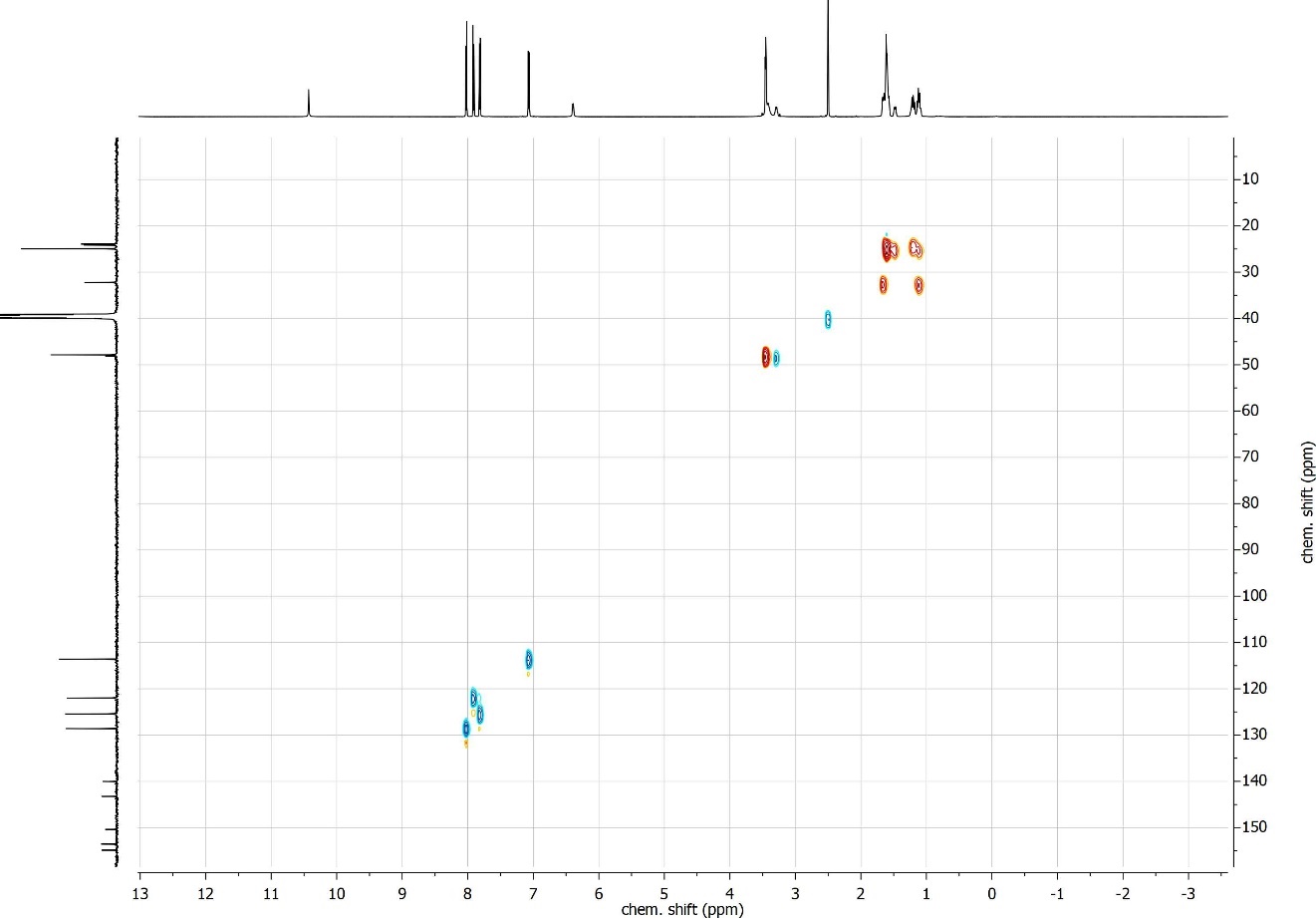
**

1. **Switching kinetics**

Supplementary Table 1: Switching kinetics.

|  | τ_on_ [s] | τ_off_ [s] |
| --- | --- | --- |
| **JB253** | 0.6660 ± 0.0202 | 0.6562 ± 0.0206 |
| **JB1793** | 0.4117 ± 0.0204 | 0.4271 ± 0.0382 |
| **JB1794** | 0.7066 ± 0.0315 | 0.5239 ± 0.0626 |
| **JB1795** | 0.5536 ± 0.0280 | 0.5388 ± 0.0055 |

## (*E*)-*N*-(Cyclohexylcarbamoyl)-4-((4-(diethylamino)phenyl)diazenyl)- benzenesulfonamide (4a or JB253)


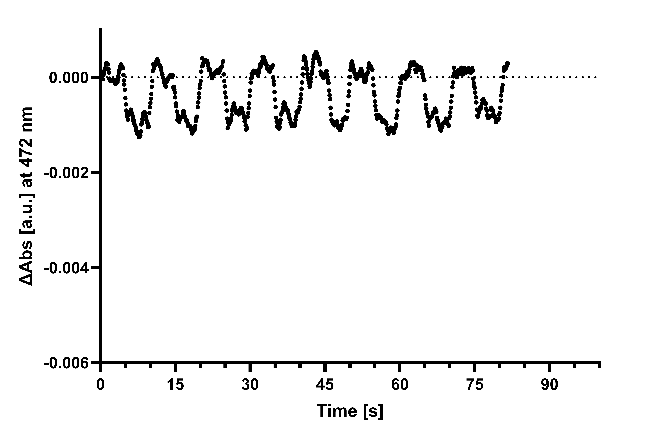


**
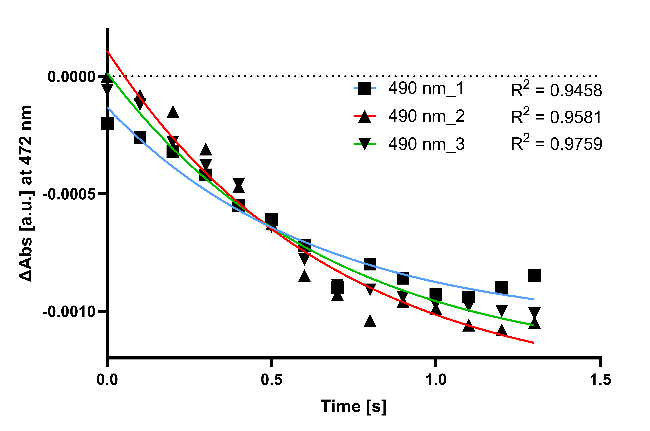
**
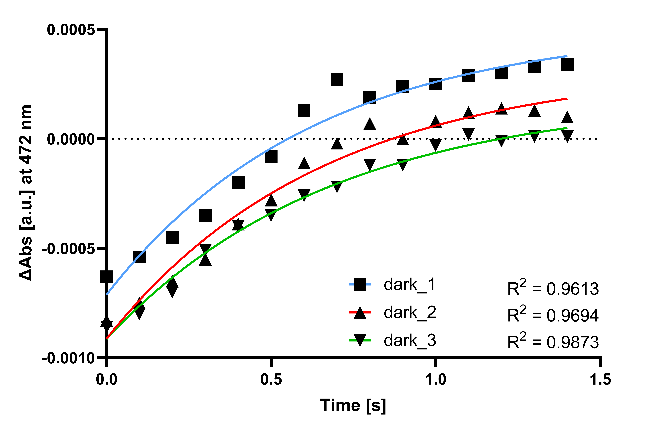


## (*E*)-*N*-(Cyclohexylcarbamoyl)-4-((4-(azetidin-1-yl)phenyl)diazenyl)- benzenesulfonamide (4b or JB1793)


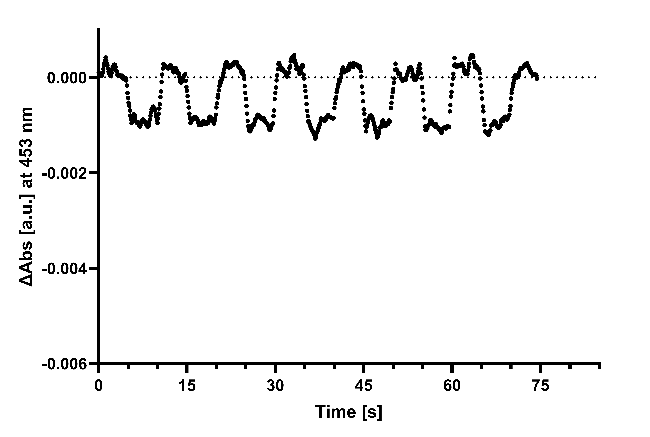


**
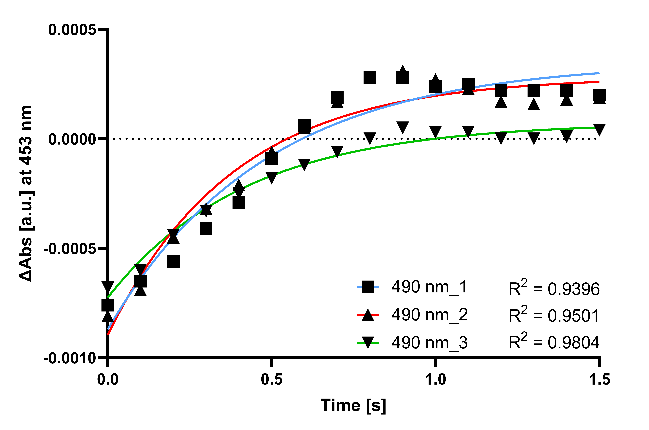

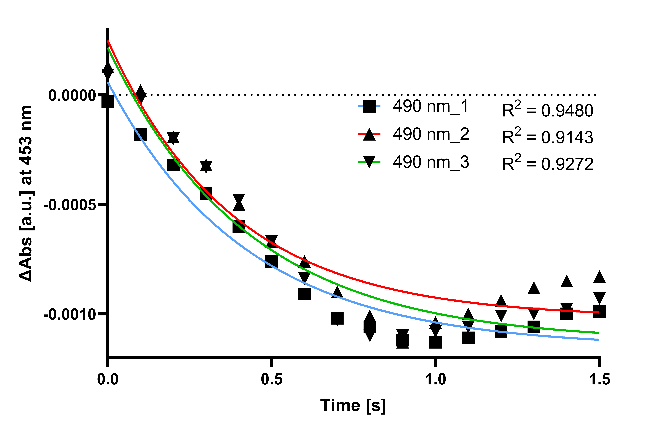
**

## (*E*)-*N*-(Cyclohexylcarbamoyl)-4-((4-(pyrrolidin-1-yl)phenyl)diazenyl)- benzenesulfonamide (4c or JB1794)

**
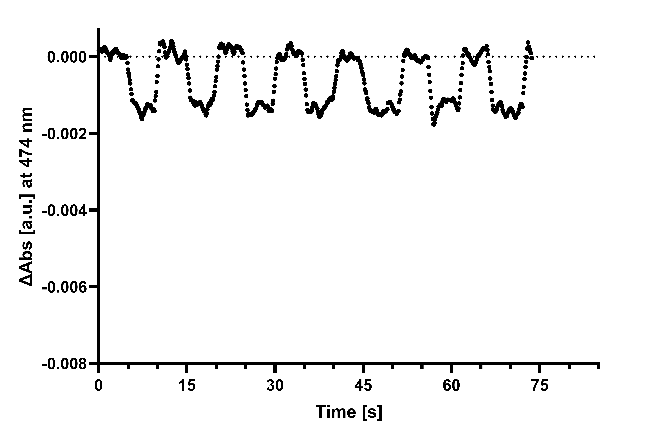
**

**
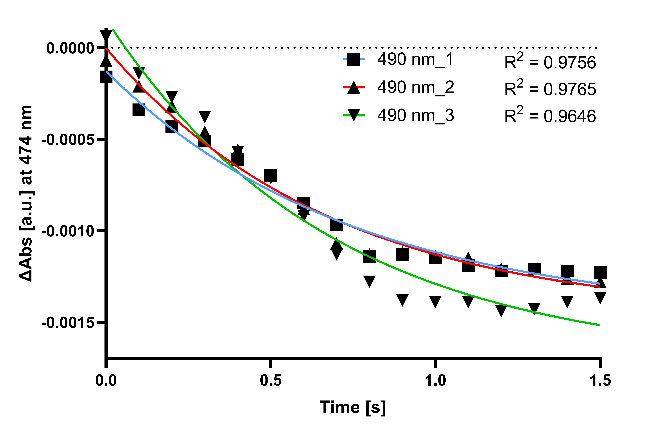

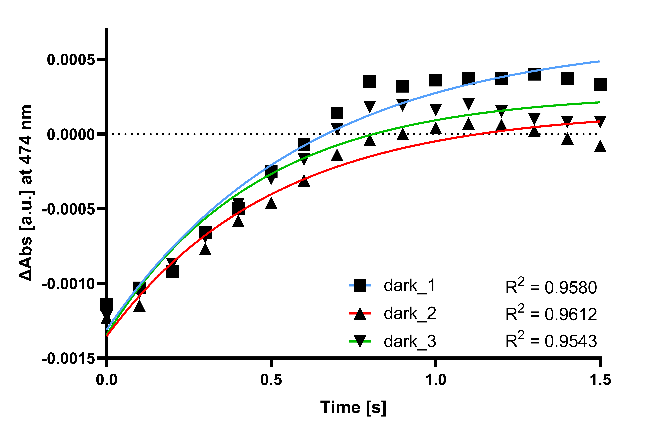
**

## (*E*)-*N*-(Cyclohexylcarbamoyl)-4-((4-(piperidin-1-yl)phenyl)diazenyl)- benzenesulfonamide (4d or JB1795)

**
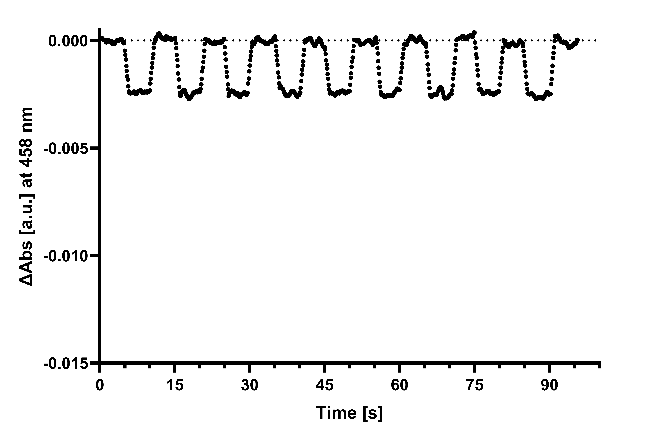
**

**
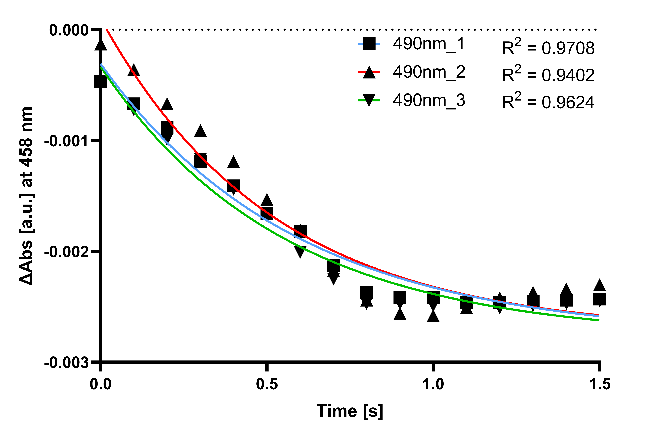

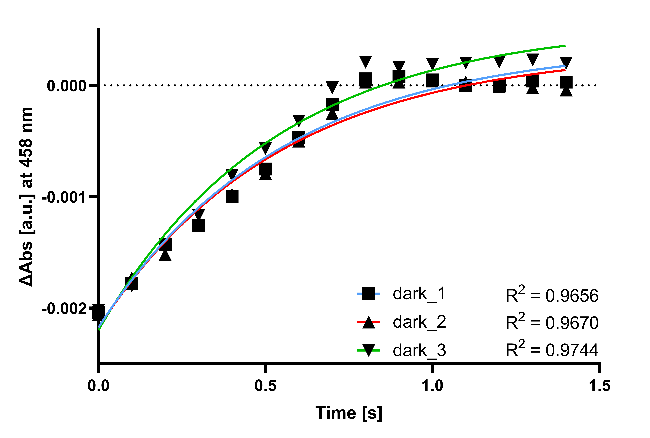
**

1. **^1^H qNMR with 1,3,5-Trimethoxybenzene as internal standard**

| **Aromatic signals** | **JB253** | **JB1793** | **JB1794** | **JB1795** |
| --- | --- | --- | --- | --- |
| Sulfonylurea | 2.000 | 1.999 | 2.004 | 1.997 |
|  | 2.002 | 2.006 | 2.004 | 1.997 |
|  | 2.000 | 2.000 | 2.000 | 2.000 |
|  | 1.989 | 1.990 | 2.004 | 1.994 |
| Internal standard | 3.661 | 3.216 | 5.839 | 3.024 |

## (*E*)-*N*-(Cyclohexylcarbamoyl)-4-((4-(diethylamino)phenyl)diazenyl)- benzenesulfonamide (4a or JB253)

**
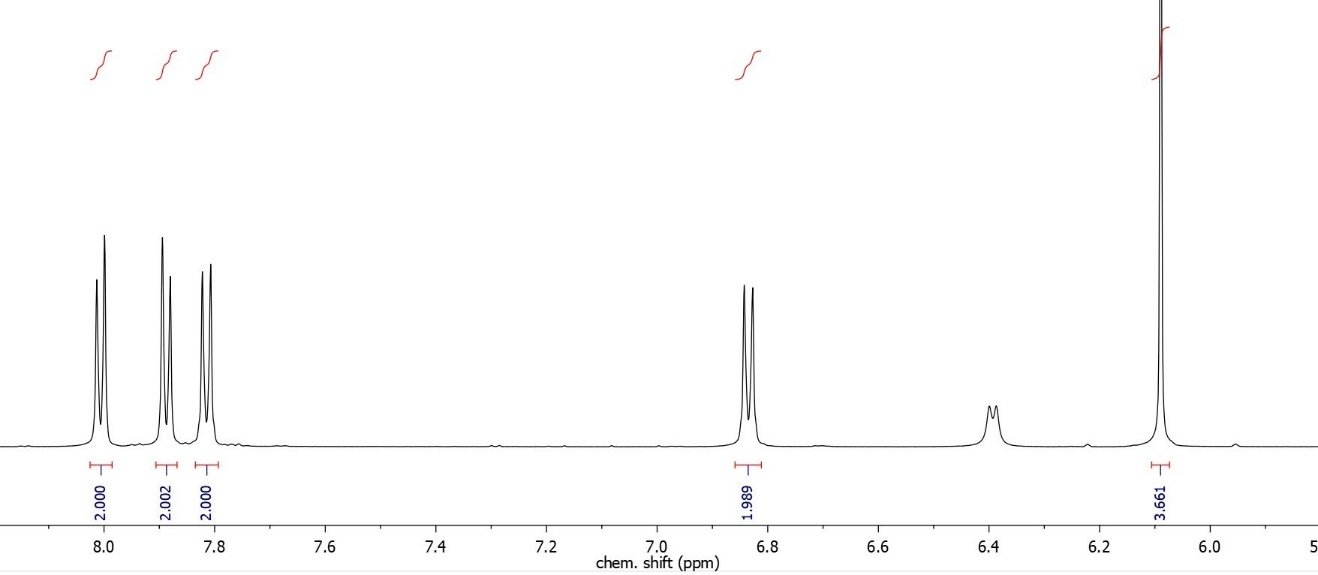
**

## (*E*)-*N*-(Cyclohexylcarbamoyl)-4-((4-(azetidin-1-yl)phenyl)diazenyl)- benzenesulfonamide (4b or JB1793)

**
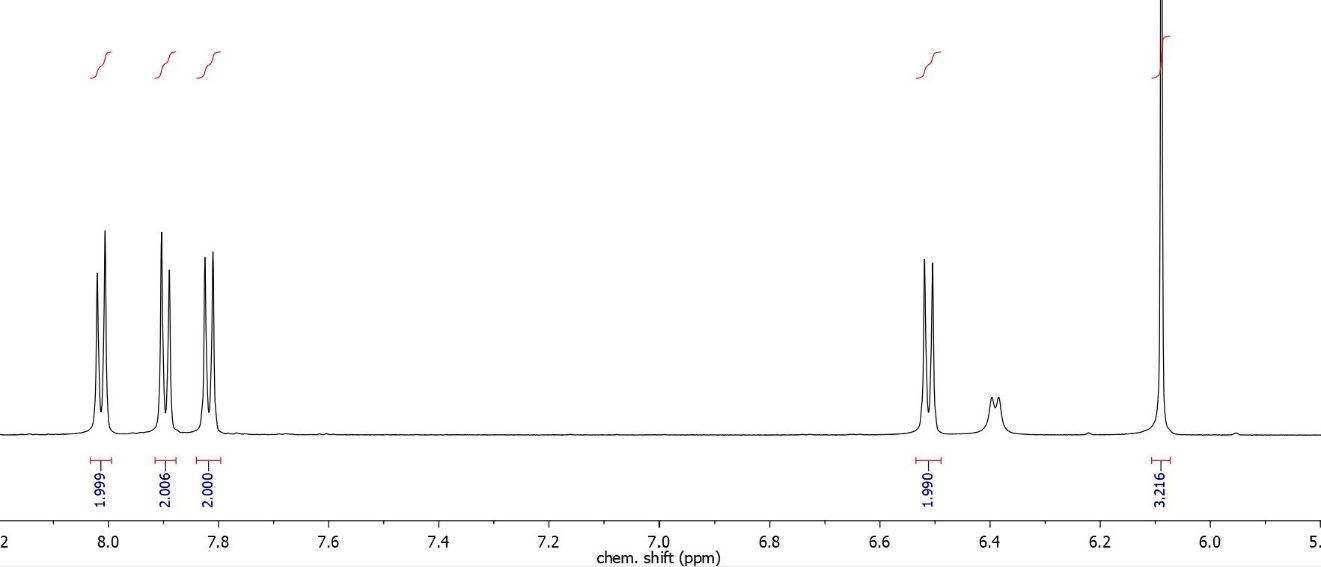
**

## (*E*)-*N*-(Cyclohexylcarbamoyl)-4-((4-(pyrrolidin-1-yl)phenyl)diazenyl)- benzenesulfonamide (4c or JB1794)


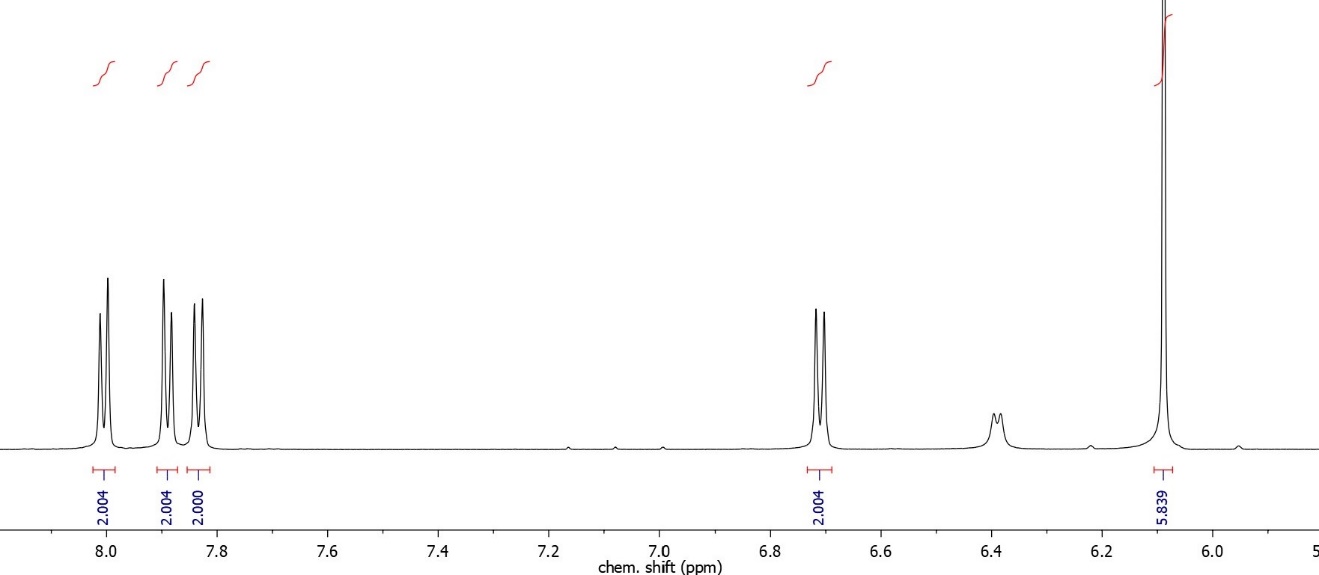


## (*E*)-*N*-(Cyclohexylcarbamoyl)-4-((4-(piperidin-1-yl)phenyl)diazenyl)- benzenesulfonamide (4d or JB1795)


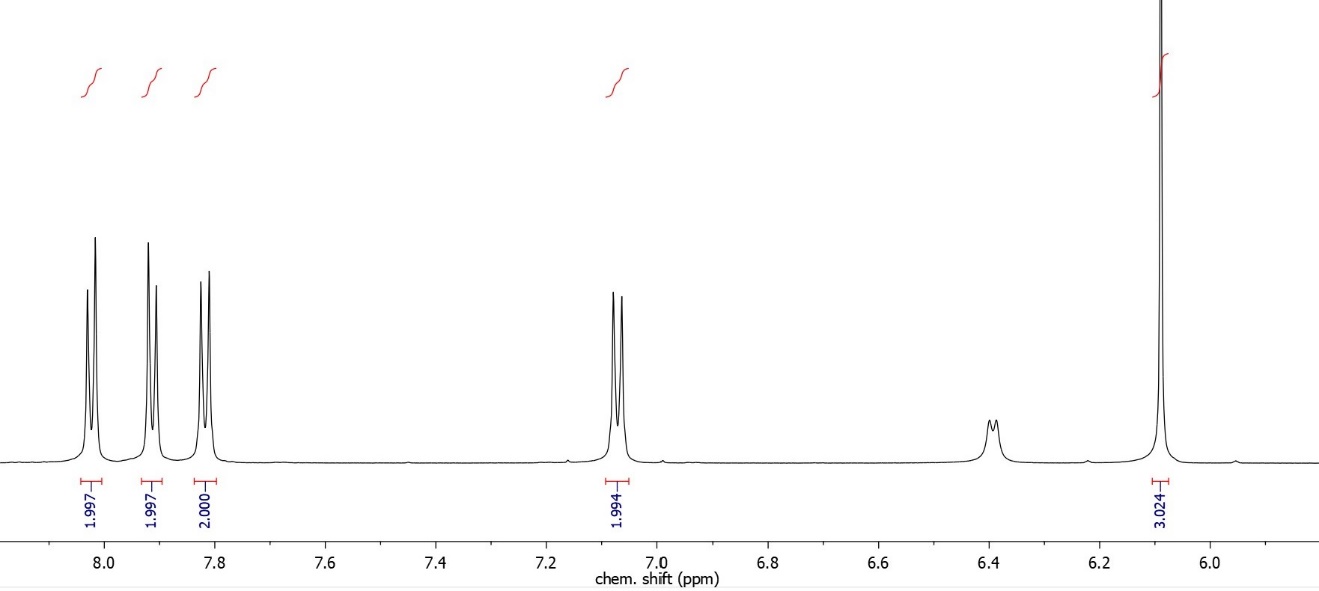


## Supporting Figure


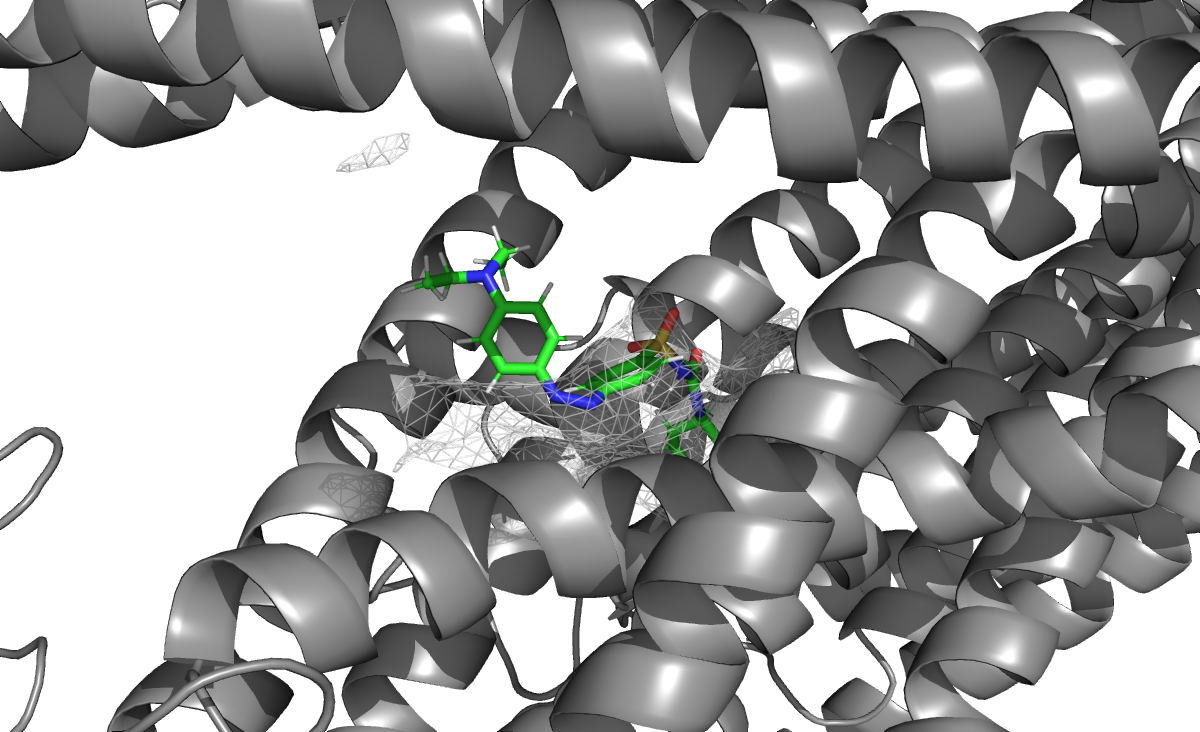


**Supporting Figure 1**: Molecular dynamics of *cis*-**JB253** bound to K_ATP_ reveals the *N*,*N*-diethylamine does not occupy many contact sites. Adopted from Walczewska-Szewc *et al.*.^2^

## References

1. Broichhagen, J. *et al.* Optical control of insulin release using a photoswitchable sulfonylurea. *Nat. Commun.* **5**, 5116 (2014).

2. Walczewska-Szewc, K. & Nowak, W. Photo-Switchable Sulfonylureas Binding to ATP-Sensitive Potassium Channel Reveal the Mechanism of Light-Controlled Insulin Release. *J. Phys. Chem. B* **125**, 13111–13121 (2021).
